# Tobacco Quitline Retreatment Interventions Among Adults With Socioeconomic Disadvantage: A Factorial Randomized Clinical Trial

**DOI:** 10.1001/jamanetworkopen.2024.43044

**Published:** 2024-11-06

**Authors:** Jesse T. Kaye, Julie A. Kirsch, Daniel M. Bolt, Kathleen H. Kobinsky, Katrina A. Vickerman, Kristina Mullis, David L. Fraser, Timothy B. Baker, Michael C. Fiore, Danielle E. McCarthy

**Affiliations:** 1 Center for Tobacco Research and Intervention, University of Wisconsin School of Medicine and Public Health, Madison; 2 Division of General Internal Medicine, Department of Medicine, University of Wisconsin School of Medicine and Public Health, Madison; 3 Institute on Aging, University of Wisconsin, Madison; 4 Department of Educational Psychology, University of Wisconsin, Madison; 5 Center for Wellbeing Research, RVO Health, Ft Mill, South Carolina

## Abstract

**Importance:**

A single round of standard tobacco quitline treatment may not be sufficient to sustain abstinence, particularly among people experiencing socioeconomic disadvantage. Adaptive retreatment may help more individuals with socioeconomic disadvantage achieve abstinence and reduce disparities in smoking cessation outcomes.

**Objective:**

To evaluate 4 evidence-based strategies for adults with limited education, no insurance, or Medicaid eligibility who continued smoking after quitline treatment.

**Design, Setting, and Participants:**

A factorial randomized clinical trial with 4 factors adapting quitline strategies was conducted for participants enrolled from June 7, 2018, to January 25, 2023, with 6-month follow-up. Adults using the Wisconsin Tobacco Quit Line who were smoking cigarettes 3 to 6 months after prior quitline treatment who were uninsured, Medicaid insured, or had no more than a high school education were included.

**Interventions:**

Quitline retreatment strategies were (1) increased counseling intensity (4 calls vs 1 call), (2) increased nicotine replacement therapy intensity (4 weeks of combination nicotine patch plus nicotine lozenge vs 2 weeks of nicotine patch), (3) text-message support (National Cancer Institute SmokefreeTXT program vs none), and (4) financial incentives for engagement in counseling and SmokefreeTXT ($30/call and/or 6-week SmokefreeTXT retention vs no incentives).

**Main Outcomes and Measures:**

Primary outcome was 7-day point-prevalence biochemically confirmed abstinence 26 weeks after the target quit day. Intention-to-treat analysis was performed.

**Results:**

Of 6019 people assessed for eligibility, 1316 (21.9%) participants were randomized (mean [SD] age, 53.1 [11.9] years; 760 [57.8%] women), and 919 (69.8%) provided final follow-up. Intention-to-treat analyses showed 162 participants (12.3%) had biochemically confirmed abstinence at 26 weeks (368 [28.0% self-reported abstinence]). There were no significant main effects for the primary outcome: 1 call (11.6% [77 of 662]) vs 4 calls (13.0% [85 of 654]) (odds ratio [OR], 1.04; 95% CI, 0.88-1.24), 2-week patch (11.2% [73 of 654]) vs 4-week combination nicotine replacement therapy (13.4% [89 of 662]) (OR, 1.12; 95% CI, 0.94-1.34), no SmokefreeTXT (13.4% [88 of 657]) vs SmokefreeTXT (11.2% [74 of 659]) (OR, 0.88; 95% CI, 0.74-1.05), and no financial incentives (12.8% [85 of 662]) vs financial incentives (11.8% [77 of 654]) (OR, 0.94; 95% CI, 0.78-1.11).

**Conclusions and Relevance:**

In this randomized clinical trial evaluating enhancements to tobacco quitlines for adults with socioeconomic disadvantage who were smoking after quitline treatment, none of the adaptive treatment strategies robustly improved long-term abstinence. Strategies are needed to enhance quitline retreatment effectiveness for adults with socioeconomic disadvantage.

**Trial Registration:**

ClinicalTrials.gov Identifier: NCT03538938

## Key Points

**Question:**

Do alterations in tobacco quitline services increase smoking abstinence among socioeconomically disadvantaged adults who did not quit after prior treatment?

**Findings:**

In this factorial randomized clinical trial including 1316 adults, treatment alterations (increased counseling, increased nicotine replacement medications, text message support, and financial incentives) did not significantly increase abstinence outcomes over standard tobacco quitline services. Overall, 12% of participants had biochemically confirmed (28% self-reported) abstinence at 26 weeks.

**Meaning:**

The findings of this study suggest that quitline enhancements did not robustly increase abstinence rates among adults with socioeconomic disadvantages who were smoking following standard quitline services.

## Teaser

This randomized clinical trial examines the use of a tobacco quitline vs more intensive interventions for adults at low socioeconomic levels who have been unable to achieve abstinence with use of quitlines alone.

## Introduction

Tobacco use remains the leading cause of death and disease in the US and disproportionately harms populations with socioeconomic disadvantages. While the prevalence of tobacco use among US adults has reached historic lows overall (11.5%), disparities have increased as tobacco use has become concentrated in disadvantaged communities.^1^ Prevalence of tobacco smoking ranges from 20% to 35% among persons who are uninsured or Medicaid eligible, have lower incomes, and have limited education (eg, high school equivalency diploma).^1^ These groups experience disproportionately greater health and economic costs related to tobacco use.^2,3^ The high rates of smoking among members of these groups do not appear to be due to low motivation to quit, as they attempt to quit at rates that are similar to those of the general population.^4^ However, these groups are less likely to use evidence-based treatment and to succeed in quitting with or without treatment.^3,4,5^ Treatment can be beneficial with such groups, however.^6,7^

Publicly funded tobacco quitlines have played a vital role in population health efforts to increase access to evidence-based smoking treatment. Quitline treatment can be useful^8^ and can be used by populations that face obstacles to using other treatment resources due to limited health insurance, transportation, and financial resources.^9^ Although quitlines are beneficial, the absolute quit rates achieved are often modest, and this is particularly true among callers with low incomes.^10,11,12^ Despite rigorous efforts, it has been difficult to enhance the effectiveness of quitline interventions among populations with low incomes.^13^ However, some approaches (eg, financial incentives for taking counseling calls) have increased treatment engagement and improved cessation outcomes^14^ in a cost-effective manner.^15^

Approaches that reengage individuals in tobacco treatment and adapt or step-up follow-up treatment for those who continue to smoke after treatment could meaningfully increase quit rates among individuals with socioeconomic disadvantage. Such retreatment strategies have been shown to increase quit rates in the general population.^16,17,18^ Moreover, there is evidence that greater quitline contact is associated with higher quit rates^19,20^; proactive reengagement may be an effective strategy for increasing such contact.

This study attempted to reengage quitline clients with socioeconomic disadvantage in additional quitline treatment if they were smoking 3 to 6 months following standard quitline treatment. A factorial experiment evaluated the effects of 4 evidence-based strategies on 26-week abstinence rates in this population. These strategies included (1) increasing the intensity of counseling (from 1 to 4 calls),^8,19,21,22^ (2) increasing the intensity of nicotine replacement therapy (NRT) (from 2 weeks of nicotine patch to 4 weeks of nicotine patch plus nicotine lozenge),^23,24,25^ (3) facilitating text-message enrollment (in the National Cancer Institute SmokefreeTXT program),^26,27,28^ and (4) providing financial incentives for engagement in smoking treatment (counseling and SmokefreeTXT).^14,29,30^ The factorial design allows for examination of the main and interactive effects so that especially effective strategies and combinations of strategies can be optimized.^31,32^

## Methods

### Experimental Design

The protocol for the trial is available in Supplement 1, and this article follows the Consolidated Standards of Reporting Trials (CONSORT) reporting guideline.^33^ The University of Wisconsin Health Sciences Institutional Review Board approved all study procedures. Participants provided informed consent and financial compensation was provided as one intervention.

In this 2 × 2 × 2 × 2 factorial optimization randomized clinical trial,^31,32^ participants were randomized to 1 of 16 treatment conditions resulting from the crossing of 4 experimental factors, each coding for a higher (on) and lesser (off) intensity: (1) quitline counseling call intensity (4 proactive calls with quit coach vs 1 call), (2) NRT intensity (4 weeks of combination nicotine patch and lozenge vs 2 weeks of patch), (3) SmokefreeTXT (encouraged to enroll in SmokefreeTXT via an SMS short code vs not given information about SmokefreeTXT), and (4) treatment engagement incentives ($30 for completing each quitline call and staying in SmokefreeTXT for 6 weeks following the quit-day vs no incentives). The primary outcome was preregistered as self-reported 7-day point-prevalence abstinence from combustible cigarettes confirmed by a negative salivary cotinine test (<10 μg/L [to convert to nanomoles per liter, multiply by 5.675]) 6 months after the target quit date (TQD) set at study enrollment. Randomization occurred in blocks of 16 (Supplement 1), stratified on participant gender (male, female, or unknown), racial minority status (minoritized racial group, White, or unknown), and socioeconomic disadvantage criterion met for study inclusion (uninsured, Medicaid insured, or no more than high school education). There were 13 stratification categories defined by the crossing of the 3 stratification variables (2 race × 2 gender × 3 socioeconomic disadvantage = 12) and 1 level for participants with unknown/other gender or race.

### Participants

The target population was 1408 English-speaking adult clients of the Wisconsin Tobacco Quit Line (WTQL) who received at least 1 counseling call with a WTQL quit coach between June 7, 2018, and March 21, 2023 (index quit attempt; eMethods in Supplement 2 includes power analysis and final sample size determination). Study eligibility required that at their index registration clients reported being uninsured, Medicaid eligible, and/or having no more than a high school education. Three to 6 months following the index quit attempt TQD, WTQL quit coaches trained in the study protocol called clients (up to 5 attempts). Enrollment inclusion criteria at this call were to reside in Wisconsin, have a unique telephone number not registered to other WTQL clients, be willing to set a new TQD within 2 weeks of enrollment, report smoking at least 6 of the past 7 days, and report smoking at least 5 cigarettes on at least 1 of the past 7 days. Prospective participants were excluded if they reported they were currently pregnant, breastfeeding, or planning to become pregnant in the next 6 months. Criteria related to smoking frequency, smoking heaviness, pregnancy, and breastfeeding were imposed to minimize risks associated with study medications. All study participants were recruited between June 7, 2018, and January 25, 2023. Recruitment was closed at 93.5% of the target sample size due to the end of the funding period.

### Procedures

#### Index Quit Attempt

The index quit attempt involved at least 1 round of standard quitline treatment that did not involve study activities. Standard WTQL treatment involved registering for WTQL services, setting a target date to quit smoking in the next 30 days, and assessing eligibility for mailed NRT (self-report or clinician-written authorization). Individuals meeting these criteria were offered 1 proactive cessation counseling call (typically completed during the registration call) and, if eligible, a 2-week supply of a single NRT (nicotine patch, gum, or lozenge). Throughout the study period, all WTQL clients had unlimited access to additional counseling by calling the WTQL. Quit coaches are available 24 hours per day, 7 days per week. However, WTQL standard protocol at the time was to offer only 1 proactive counseling call, 2 weeks of single NRT (once per year), and not to offer text message service to all clients. During this study it was standard practice to obtain WTQL client consent during the registration process for follow-up contacts to assess treatment effectiveness.

#### Research Procedures

Study outreach occurred 3 to 6 months after the index quit attempt TQD. A research-trained WTQL quit coach proactively called clients who met eligibility prerequisites to assess their current smoking status. For clients reporting smoking in the past 7 days at this follow-up contact, the quit coach described the study and invited the client to complete phone screening to assess study eligibility. Eligible individuals completed an oral consent process that described study procedures, risks, and benefits. Individuals who consented confirmed their WTQL registration data and completed a baseline assessment before being randomized and informed about what treatment they would receive. These procedures were completed in a single call whenever possible. Individuals who declined or were ineligible to participate were offered another round of standard WTQL services. In October 2020, the WTQL began offering intensive treatment (including 7 proactive calls and 12 weeks of combination NRT) to clients who identified as American Indian or Alaska Native at intake and began offering text messaging support to clients aged 18 to 24 years. The availability of these alternatives to study participation were described in the informed consent process after these programs were launched.

Study interventions were delivered by existing WTQL quit coaches and medication fulfillment teams. As such, the counseling protocols and medication eligibility screening and fulfillment processes used in the study followed standard practice at the WTQL. Participants did not have to be eligible for NRT to enroll in the study. Treatment incentives were delivered via gift cards mailed by the WTQL. The only study-specific modification to standard WTQL practice during the study was adding a 1-page study flyer to standard WTQL mailings to newly registered clients, beginning in December 2019.

Following enrollment, the contact information for randomized participants was sent securely by the WTQL to the research team. The research team mailed a welcome packet to enrolled participants containing a study information sheet that described the intervention components available to them.

The research team attempted phone interviews with participants 12 and 26 weeks after their TQD. Those who reported no smoking in the past 7 days at the 26-week follow-up were asked to provide a mailed saliva sample for cotinine testing to validate reported abstinence. The research team mailed collection kits (Forensic Fluids Laboratories) with instructions and offered recipients phone support for sample collection and prepaid return. Participants were compensated for follow-up ($30/call, $40 for returning saliva sample).

Registration data regarding client-reported demographic characteristics, educational level, insurance type, tobacco use, time to first cigarette after waking, quitting motivation, chronic medical conditions, and 3-digit zip code prefix were collected at baseline by the WTQL at both the index WTQL treatment episode and study enrollment. Race and ethnicity and other assessments were self-reported by clients at the time of quitline registration, using the classifications in the minimum dataset intake requirements specified by the North American Quitline Consortium for US quitlines.^34^ Additional baseline data were collected at enrollment regarding menthol use, smoking history, psychiatric history, economic factors, and texting use (Table 1). Area Social Deprivation Index scores^35^ associated with participants’ baseline addresses were collected from public data sources.

**Table 1.** Characteristics of 1316 Participants

Variable^a^	No. (%)^b^
Gender (n = 1312)	
Female	760 (57.8)
Male	552 (41.9)
Ethnicity (n = 1308)^c^	
Hispanic or Latino	49 (3.7)
Not Hispanic or Latino	1259 (95.7)
Race (n = 1308)^c^	
Black or African American	349 (26.5)
White	866 (65.8)
Other	93 (7.1)
Health insurance plan type (n = 1311)	
Commercial	64 (4.9)
Medicaid	756 (57.4)
Medicare	238 (18.1)
Uninsured	253 (19.2)
Education (n = 1308)	
Less than grade 12/GED	292 (22.2)
Grade 12 or GED	575 (43.7)
Some college or more	441 (33.5)
Employment (n = 1305)	
Unable to work/disabled	557 (42.3)
Employed	414 (31.5)
Not employed for wages	176 (13.4)
Retired	158 (12.0)
Tobacco product use (n = 1311)	
Cigarette use only	1127 (86.0)
Cigarette and other tobacco product use	184 (14.0)
Smokes menthol cigarettes (n = 1311)	
No	627 (47.6)
Yes	684 (52.0)
Time to use tobacco after waking (n = 1301)	
Within 30 min	1118 (85.6)
After 30 min	188 (14.4)
Has a chronic condition (n = 1311)^d^	766 (58.4)
Ever treated for depression (n = 1305)	707 (53.7)
Ever treated for anxiety (n = 1309)	634 (48.2)
Ever treated for bipolar disorder (n = 1308)	291 (22.1)
Ever treated for schizophrenia (n = 1307)	124 (9.4)
Age, mean (SD), y (n = 1316)	53.1 (11.9)
Motivation to quit smoking, 1-10, mean (SD) (n = 1080)^e^	8.5 (1.7)
Cigarettes per day, 0-99, mean (SD) (n = 1265)	18.2 (10.4)
Total income from previous year, mean (SD), $ (n = 1112)	24 722 (23 530)
Social Deprivation Index score, mean (SD) (n = 1296)^f^	54.0 (28.9)

Abbreviation: GED, general educational development.

^a^ All demographic, smoking, and health characteristics were self-reported by participants at study enrollment.

^b^ Percentages based on total population (n = 1316) vs subgroups.

^c^ Race and ethnicity was self-reported by clients at the time of quitline registration using the classifications in the Minimum Data Set Intake requirements specified by the North American Quitline Consortium for US quitlines. Other race includes Arab or Arab American (n = 2), Asian (n = 3), American Indian/Alaska Native (n = 14), Native Hawaiian/Pacific Islander (n = 2), Multiracial (n = 6), and self-reported Other response option (n = 66).

^d^ Chronic conditions included asthma, diabetes, chronic obstructive pulmonary disease, or cardiovascular disease.

^e^ Motivation to quit smoking rated from 1, not at all, to 10, extremely.

^f^ Social Deprivation Index score is a composite measure ranging from 1 to 100, with higher scores reflecting greater severity of deprivation.

At the 12- and 26-week follow-ups, interviewers assessed the primary outcome (smoking in the past 7 days), use of smoking treatment, use of other tobacco or nicotine products and associated out-of-pocket spending, quitting motivation and confidence, and smoking triggers and motives.^36^ At the 12-week follow-up, additional variables were assessed regarding satisfaction with treatment elements (eg, quit coaching and medication). Health care use and days of work or school missed due to illness were assessed at the 26-week follow-up.

In addition to self-reported data on study treatment engagement, WTQL and SmokefreeTXT records regarding treatment delivery were collected (eTable 2 in Supplement 2). Data on the number of counseling calls completed and medication dispensing were provided by WTQL. SmokefreeTXT provided records regarding registration data, program disengagement (when a user texted stop within the first 6 weeks post-TQD), and responses to texted prompts to report smoking status, craving, mood, and perceived difficulty over first 6 weeks of the program.

###  Statistical Analysis 

The primary analysis examined abstinence rates to identify the intervention components and combinations with significant main and interactive effects on quitting success. The primary abstinence end point was biochemically verified 7-day point-prevalence abstinence at 26 weeks after TQD. Secondary abstinence outcomes were self-reported 7-day point-prevalence abstinence 12 and 26 weeks after TQD. In primary analyses, we used an intention-to-treat approach in which all missing cases are analyzed and we coded missing cases as nonabstinent (including those lacking biochemical verification). We supplemented this with sensitivity analyses under different assumptions regarding missing data (ie, multiple imputation missing at random and missing not at random, assuming missing data decreases the log-odds of abstinence by 1.0) (Supplement 1). Treatment main and interaction effects on binary abstinence end points were analyzed using logistic regression with effect coding (with levels coded −1 for control and 1 for each quitline enhancement). We planned to test all 2-, 3-, and 4-way interactions, although only three 2-way interactions were hypothesized a priori (significance level *P* < .05 with 2-sided, unpaired testing); these intervention combinations were expected to especially increase abstinence: incentives with increased counseling calls, incentives with SmokefreeTXT, and increased counseling calls with increased NRT intensity. Covariates included 12 binary-coded variables representing the observed randomization stratification categories with the most frequent category (ie, female, White, Medicaid insured) used as the reference condition. Sensitivity analyses examined models adjusted for a priori selected covariates: age, Social Deprivation Index score, and longest past quit attempt. Analysis was conducted using SPSS, version 29.9.2.0 (IBM Statistics) and R, version 4.4.1 (R Foundation for Statistical Computing).^37^

## Results

### Participant Characteristics

Table 1 depicts summary statistics for the 1316 enrolled participants (mean [SD] age, 53.1 [11.9] years; 760 [57.8%] women; 552 [41.9%] men; 1259 [95.7%] not Hispanic or Latino; 349 [26.5%] Black or African American; and 866 [65.8%] White). eTable 1 in Supplement 2 depicts summary statistics by the 4 experimental factors.

### Engagement and Retention

**Figure 1.**
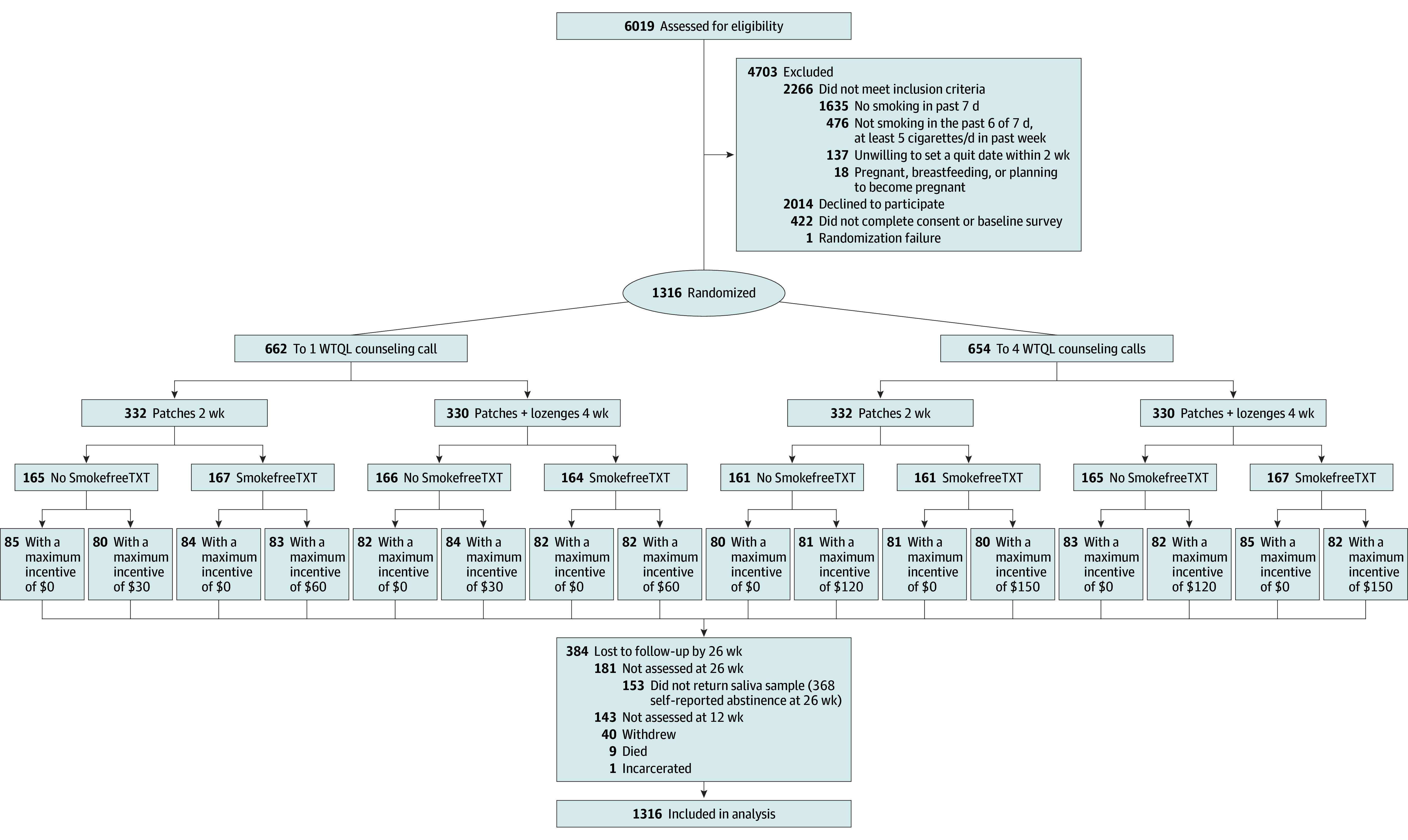
Participant Flow Diagram Participants randomized to 1 level (“on” vs “off”) of 4 orthogonal factors. A total of 384 Individuals were lost to follow-up by 26 weeks, and 368 individuals self-reported abstinence at 26 weeks. WTQL indicates Wisconsin Tobacco Quit Line.

Figure 1 shows a modified CONSORT diagram. Because of the complexity of the design, data are not presented for each of the 16 randomized treatment combinations. Participants reported very high motivation to quit (mean [SD], 8.5 [1.7] on a 1- to 10-point scale). Attrition was low overall and did not differ substantially by treatment factor. The 26-week follow-up call was completed by 1075 (81.7%) participants, with 368 (28.0%) reporting abstinence. Rates of returning saliva samples for cotinine testing were low; only 212 of 368 participants (57.6%) who reported abstinence 26 weeks after TQD provided samples (156 [42.4%] who reported abstinence did not return saliva samples). Rates did not differ substantially by condition. Therefore, a total of 919 participants (69.8%) provided primary outcome 26-week follow-up data (707 self-reported smoking, 212 saliva sample). Analyses of intervention delivery (eTable 2 in Supplement 2) confirmed that those in the 4-call condition received more calls than those in the 1-call condition (mean [SD], 2.7 [1.6] vs 1.2 [1.5] calls), only those in the combination NRT condition received nicotine lozenges (87.4% received lozenges), only those in the incentive condition received incentives (mean [SD], $52.70 [$36.72] per participant), and only those in the SmokefreeTXT condition enrolled via the study short code (19.3% enrolled).

#### Abstinence

**Figure 2.**
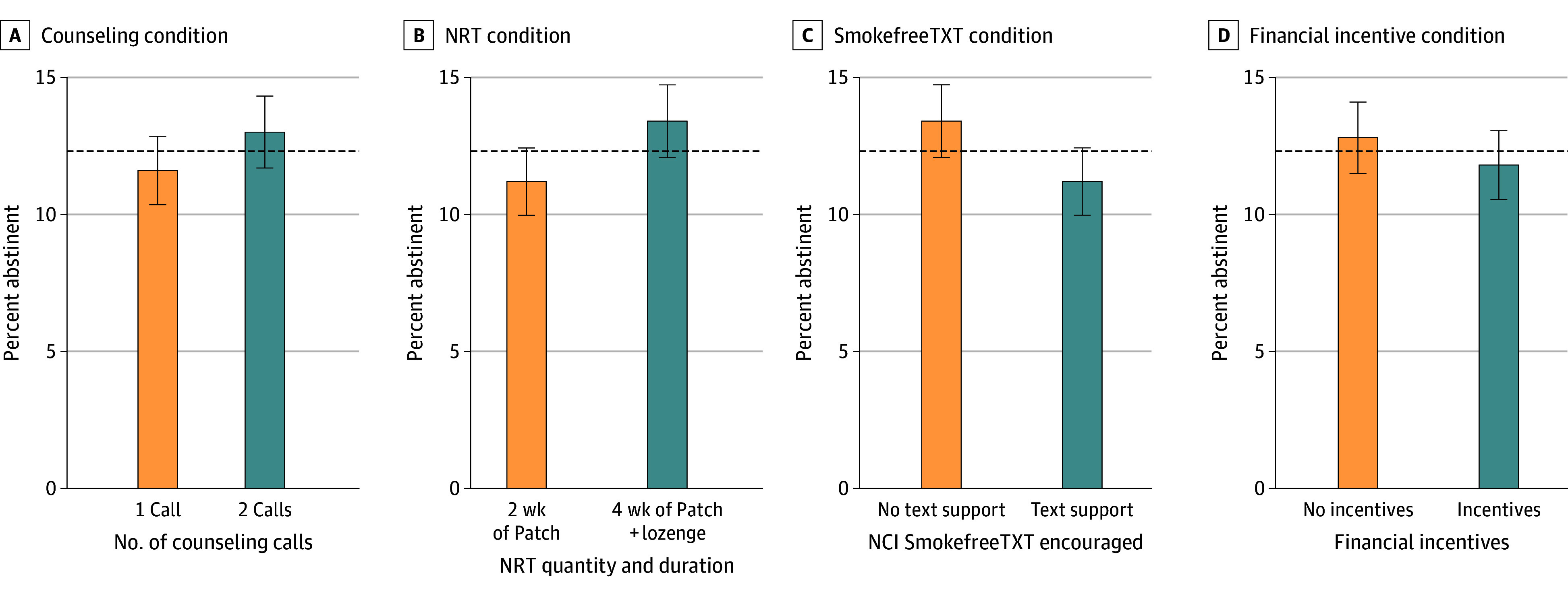
Biochemically Confirmed Intention-to-Treat Abstinence Rates 26 Weeks After Target Quit Day by Treatment Condition Primary outcome of 26-week biochemically verified abstinence representing the effects of each of the 4 factors. Error bars represent approximate SEs, defined as √(p × [1 − p] / n), where p is the percent abstinent and n is the number of participants who contributed to the bar. Error bars are approximate given the assumption of independent observations. Factorial data were modeled with higher-order interactions so all lower-order effects are dependent on the level of the other factors. Dashed line represents the mean biochemically confirmed intention-to-treat abstinence rate (12.3%) across all conditions. NCI indicates National Cancer Institute; NRT, nicotine replacement therapy.

Intention-to-treat 7-day point-prevalence abstinence rates confirmed by salivary cotinine testing at 26 weeks after TQD are displayed by the 4 factors in Figure 2 (eFigure 1 in Supplement 2 displays all 16 conditions). Overall, 12.3% of participants (162 of 1316) had biochemically confirmed abstinence at 26 weeks (397 missing). There were no significant main effects: primary abstinence outcomes were 1 call (11.6% [77 of 662]) vs 4 calls (13.0% [85 of 654]) (OR, 1.04; 95% CI, 0.88-1.24), 2-week patch (11.2% [73 of 654]) vs 4-week combination with NRT (13.4% [89 of 662]) (OR, 1.12; 95% CI, 0.94-1.34), no SmokefreeTXT (13.4% [88 of 657]) vs SmokefreeTXT (11.2% [74 of 659]) (OR, 0.88; 95% CI, 0.74-1.05), and no financial incentives (12.8% [85 of 662]) vs financial incentives (11.8% [77 of 654]) (OR, 0.94; 95% CI, 0.78-1.11). There were no significant 2-way interactions, but there was a significant 4-way interaction (Wald = 4.22; *P* = .04) and a significant 3-way interaction of incentives × NRT × counseling calls (Wald = 6.07; *P* = .01) (Table 2; eFigure 2 in Supplement 2, the highest abstinence rate [17.1%] observed with the combination of 4 counseling calls, combination NRT, and financial incentives).

**Table 2.** Logistic Regression Models for 26 Weeks Biochemically Verified 7-Day Point Prevalence Abstinence^a^

Test	B (SE)	Wald	*P* value	OR (95% CI)
Main effects				
Counseling	0.042 (0.090)	0.22	.64	1.04 (0.88 to 1.24)
NRT	0.118 (0.090)	1.745	.19	1.13 (0.94 to 1.34)
SmokefreeTXT	−0.127 (0.090)	2.02	.16	0.88 (0.74 to 1.05)
Financial incentive	−0.068 (0.090)	0.57	.45	0.94 (0.78 to 1.11)
2-Way interactions				
Counseling × NRT	0.057 (0.090)	0.41	.52	1.06 (0.89 to 1.26)
Counseling × SmokefreeTXT	0.013 (0.090)	0.02	.89	1.01 (0.85 to 1.21)
Counseling × incentive	0.023 (0.090)	0.09	.77	1.03 (0.86 to 1.22)
NRT × SmokefreeTXT	−0.111 (0.090)	1.54	.22	0.90 (0.75 to 1.07)
NRT × incentive	0.038 (0.090)	0.18	.67	1.04 (0.87 to 1.24)
SmokefreeTXT × incentive	−0.070 (0.090)	0.60	.44	0.93 (0.78 to 1.11)
3-Way interaction				
Counseling × NRT × SmokefreeTXT	0.013 (0.090)	0.02	.89	1.01 (0.85 to 1.21)
Counseling × NRT × incentive	0.221 (0.090)	6.07	.01	1.25 (1.05 to 1.49)
Counseling × SmokefreeTXT × incentive	−1.54 (0.090)	2.93	.09	0.86 (0.72 to 1.02)
NRT × SmokefreeTXT × Incentive	0.083 (0.090)	0.86	.36	1.09 (0.99 to 1.30)
4-Way interaction				
Counseling × NRT × SmokefreeTXT × incentive	0.184 (0.090)	4.22	.04	1.20 (1.01 to 1.43)

Abbreviations: NRT, nicotine replacement therapy; OR, odds ratio.

^a^ Intention-to-treat analyses were used including all participants in the analysis under the assumption that missing data reflect smoking (coded as 0 = smoking, 1 = abstinent). Effects coding was used for the main effects and all higher-order interactions (levels coded as −1 for control and 1 for active enhancement). Model covariates include 12 binary-coded variables representing the observed stratification categories (the most frequent category [female, White, and Medicaid insured] was used as the reference); the model was not adjusted for any other covariates.

We conducted sensitivity analyses to evaluate the robustness of results with regard to assumptions made about the missing abstinence data. These alternative assumptions included missing at random using multiple imputation and a missing-not-at-random condition in which the log-odds of abstinence under missing at random was reduced by a value of 1 for missing outcomes.^38^ No main effects or 2-way interactions emerged in either sensitivity analysis. The interactions were also significant under the missing-not-at-random condition (eTable 4 in Supplement 2), but the 4-way interaction was not significant under the missing-at-random condition (eTable 5 in Supplement 2). An intention-to-treat sensitivity analysis adjusted for the a priori covariates age, Social Deprivation Index score, and longest past quit attempt yielded results similar to those of the main model (eTable 6 in Supplement 2). Likewise, a similar pattern of results emerged in models using 26-week self-reported point-prevalence abstinence (eg, the 3- and 4-way interactions were found), although overall abstinence rates were higher (mean across conditions, 28.0% [368 of 1316], 241 missing) (eTable 3 in Supplement 2). Only 44% of participants (162 of 368) who self-reported 26-week abstinence provided saliva samples negative for cotinine that biochemically verified abstinence.

Overall, 25.5% of randomized participants reported 7-day point-prevalence abstinence rates at 12 weeks after TQD. There was a significant main effect of counseling intensity (OR, 1.15; 95% CI, 1.02-1.31; Wald = 4.78; *P* = .03; 1 counseling call, 23.0%; 4 counseling calls, 28.1%), and no other significant main effects or higher-order interactions (Table 3).

**Table 3.** Logistic Regression Models for Secondary Outcome of 12 Weeks Self-Reported 7-Day Point Prevalence Abstinence^a^

Test	B (SE)	Wald	*P* value	OR (95% CI)
Main effects				
Counseling	0.141 (0.065)	4.78	.03	1.15 (1.02 to 1.31)
NRT	0.039 (0.065)	0.37	.54	1.04 (0.92 to 1.18)
SmokefreeTXT	−0.013 (0.065)	0.04	.84	0.99 (0.87 to 1.12)
Financial incentive	−0.065 (0.065)	1.03	.31	0.94 (0.83 to 1.06)
2-Way interactions				
Counseling × NRT	0.044 (0.065)	0.48	.49	1.05 (0.92 to 1.19)
Counseling × SmokefreeTXT	−0.048 (0.065)	0.56	.46	0.95 (0.84 to 1.08)
Counseling × incentive	−0.068 (0.065)	1.13	.29	0.93 (0.82 to 1.06)
NRT × SmokefreeTXT	−0.001 (0.065)	0.00	.99	1.00 (0.88 to 1.13)
NRT × incentive	−0.022 (0.065)	0.11	.74	0.98 (0.86 to 1.11)
SmokefreeTXT × incentive	0.117 (0.065)	3.27	.07	1.12 (0.99 to 1.28)
3-Way interaction				
Counseling × NRT × SmokefreeTXT	−0.087 (0.065)	1.80	.19	0.92 (0.81 to 1.04)
Counseling × NRT × incentive	0.038 (0.065)	0.35	.56	1.04 (0.92 to 1.18)
Counseling × SmokefreeTXT × incentive	−0.088 (0.065)	1.85	.17	0.92 (0.81 to 1.04)
NRT × SmokefreeTXT × incentive	−0.005 (0.065)	0.006	.94	0.99 (0.88 to 1.13)
4-Way interaction				
Counseling × NRT × SmokefreeTXT × incentive	−0.091 (0.065)	1.97	.16	0.91 (0.81 to 1.04)

Abbreviations: NRT, nicotine replacement therapy; OR, odds ratio.

^a^ Intention-to-treat analyses were used including all participants in the analysis under the assumption that missing data reflect smoking (coded as 0 = smoking, 1 = abstinent). Effects coding was used for the main effects and all higher-order interactions (levels coded as −1 for control and 1 for active enhancement). Model covariates include 12 binary-coded variables representing the observed stratification categories (the most frequent category [female, White, and Medicaid insured] was used as the reference); the model was not adjusted for any other covariates.

## Discussion

Participants in this research were adults with limited education, Medicaid, or no insurance who were smoking 3 to 6 months after tobacco quitline treatment. These individuals were offered an opportunity to reengage in quitline treatment that could involve up to 4 experimental strategies intended to increase quitline treatment effectiveness. Main effects tests showed that none of the interventions produced a significant main effect on biochemically confirmed abstinence at 6-month follow-up, using self-report or biochemically confirmed outcomes (Table 2). Increasing the number of counseling phone calls improved self-reported abstinence rates at 12-week follow-up (Table 3), but this effect was not maintained at 6 months (eTable 3 in Supplement 2). Although offering more counseling calls generally increases quitting success,^22^ other recent research has also not identified significantly more effective quitline treatments delivered to individuals with low income^13^; these populations may have intersecting vulnerabilities that contribute to treatment challenges (eg, co-occurring conditions). Despite very high motivation to quit (mean [SD], 8.5 [1.7] on a 1-10 scale), most participants were unable to do so. Therefore, improving tobacco treatment engagement and effectiveness for this population remains a pressing and unresolved public health imperative.

Some significant higher-order interactions were found at 26 but not 12 weeks. These interactions were not predicted and may reflect chance associations. Examination of the interactions suggests that the combination of incentives, 4 counseling calls, and combination NRT tended to produce relatively good abstinence outcomes (eFigure 2 in Supplement 2). However, inspection of condition differences that contribute to the 3- and 4-way interactions did not reveal a consistent pattern of effects. There is a tendency for combining multiple intervention components to erode the effectiveness of the co-occurring components,^39^ and this may have suppressed the effectiveness of some intervention combinations. While the combination of extended counseling, combination NRT, and incentives for treatment engagement tended to produce relatively high abstinence rates (17.1%), the small number of individuals receiving only this combination (n = 164) precludes well-powered tests focused on its effectiveness. Future studies could examine increasing the intensity of these strategies (eg, to at least 8 weeks of NRT), as currently implemented in some state quitlines. The current study does not speak to the potential effectiveness of such increases.

This research suggests that systematic efforts to reengage individuals with socioeconomic disadvantage in quitline treatment may meaningfully increase long-term quit rates. When averaged across combinations of intervention components, intention-to-treat 6-month abstinence rates ranged from 12.3% (biochemically verified) to 28.0% (self-reported). Even a 12.3% abstinence rate would be clinically meaningful given the cessation challenges faced by this population. However, it is unclear whether these encouraging abstinence rates were due to retreatment per se as the study lacked an untreated control group and various research features (eg, screening criteria, consent requirement) might have biased the sample.

### Limitations

This trial has limitations. A large percentage (42.4%) of participants reporting abstinence at 6 months did not provide samples for biochemical confirmation. However, similar patterns of results were observed for self-reported abstinence (eTable 3 in Supplement 2) and there were no systematic differences in biochemical confirmation across conditions. Second, there was very low engagement in SmokefreeTXT services (19.3% enrolled, 74.0% participated at least 6 weeks); thus, our results speak more to the reach of SmokefreeTXT via passive referral rather than its effectiveness. Future studies could examine automatic enrollment into a text program. Similarly, participants eligible for 4 proactive counseling calls did not complete all available calls; as such, our results may underestimate the effectiveness of such a counseling regimen with greater engagement. In addition, people who smoked less (<5 cigarettes/d) or intermittently (nondaily) were excluded from the study, which limits the generalizability of our findings since these patterns are prevalent among people with lower incomes.

## Conclusions

In this factorial randomized clinical trial, adults with socioeconomic disadvantage who did not quit smoking with standard quitline treatment achieved encouraging abstinence rates when they were proactively reengaged in quitline treatment. Four promising strategies intended to enhance the effectiveness of repeat quitline treatment (more counseling calls, more NRT, the National Cancer Institute SmokefreeTXT program, and financial incentives) did not produce statistically significant main effects on long-term abstinence rates in this study. Increasing counseling calls increased self-reported abstinence 12 weeks after quitting, but not at 26 weeks. At 26 weeks, higher-order interactions reflected inconsistent effects of quitline enhancement components. These results highlight the potential value of offering retreatment to quitline clients with socioeconomic disadvantages and point to a need to test other strategies to enhance retreatment effectiveness in this population.

## Notes

Supplement 1.
Trial Protocol

Supplement 2. **eTable 1.** Participant Demographic and Tobacco Use Characteristics by Four Experimental Factors and Overall **eTable 2.** Intervention Delivery by Condition and by Factor Level in the 2x2x2x2 Factorial Experiment (N = 1316) **eTable 3.** Logistic Regression Models for 26-Week Post-Quit-Date Self-Reported 7-Day Point Prevalence Abstinence Using the Intent-to-Treat Principle With Missing Cases Coded as Smoking **eTable 4.** Sensitivity Analysis of Logistic Regression Models for Primary Outcome 26-Week Post-Quit-Date Biochemically Verified 7-Day Point Prevalence Abstinence Using Missing-Not-at-Random (MNAR) **eTable 5.** Sensitivity Analysis of Logistic Regression Models for Primary Outcome 26-Week Post-Quit-Date Biochemically Verified 7-Day Point Prevalence Abstinence Using Multiple Imputation (Missing-at-Random) **eTable 6.** Sensitivity Analysis of Adjusted Logistic Regression Models for Primary Outcome 26-Week Post-Quit-Date Biochemically Verified 7-Day Point Prevalence Abstinence Using the Intent-to-Treat Principle With Missing Cases Coded as Smoking **eFigure 1.** Biochemically Confirmed Intent-to-Treat 26-Week Post-Quit-Day Abstinence Rates by All Treatment Combinations **eFigure 2.** Biochemically Confirmed Intent-to-Treat 26-Week Post-Quit-Day Abstinence Rates by Counseling Calls x NRT x Financial Incentives
eReferences

Supplement 3.
Data Sharing Statement
