# Supplementary material for: Tobacco Quitline Retreatment Interventions Among Adults With Socioeconomic Disadvantage: A Factorial Randomized Clinical Trial
Source: JAMA Netw Open. 2024 Nov 6;7(11):e2443044. doi: 10.1001/jamanetworkopen.2024.43044 (PMC11541633; doi:10.1001/jamanetworkopen.2024.43044)
Supplement: Supplement 1. — Trial Protocol [file jamanetwopen-e2443044-s001.pdf]

Title: Research Plan and Protocol: Improving Quitline Support (IQS) Study

Principal Investigators: Danielle E. McCarthy (Contact PI) and Michael C. Fiore, University of Wisconsin School of Medicine and Public Health, Department of Medicine, Center for Tobacco Research and Intervention

Protocol Version: September 20, 2022

Coordinating Center Identification: University of Wisconsin Center for Tobacco Research and Intervention

Co-Investigators: Timothy B. Baker (University of Wisconsin School of Medicine and Public Health)

Study Coordinator: Kate Kobinsky

Funding Sponsor: National Cancer Institute, National Institutes of Health, Grant R01CA218156

Research Plan and Protocol: Improving Quitline Support (IQS) Study

I. SIGNIFICANCE

Cigarette smoking is the leading preventable cause of cancer and many other deadly diseases, and the burden of tobacco use is greatest among those with limited socioeconomic resources[1]. This proposal focuses on low-income smokers (i.e., Medicaid-eligible and uninsured) because smoking prevalence in these groups remains very high (27-30%), both in absolute terms and relative to the general population[2-4]. Low-income smokers also develop cancer at higher rates[5] and struggle more to quit than do higher-income smokers[6-10]. These disparities in tobacco use and its burdens must be addressed. Highly effective cessation treatment services that can be delivered remotely, flexibly, and conveniently with few cost or logistical barriers have the potential to achieve this aim.

The Wisconsin Tobacco Quit line (WTQL) is a public (state-sponsored) program that serves residents of Wisconsin who smoke, including smokers with little education and uninsured and low-income residents eligible for Medicaid (BaderCare Plus in Wisconsin), a public health insurance program. This proposal focuses on Medicaid-eligible, uninsured, and low-education smokers because smoking prevalence in these groups is roughly double that of the general population[2-4]. The proposal focuses on enhancing care delivered through the WTQL, a low-barrier service with broad reach and demonstrated attractiveness in this population; about half of callers to tobacco quit lines in Wisconsin and in the U.S. report being uninsured or covered by Medicaid[11-12], and still more have no more than a high school education, which is often associated with socioeconomic disadvantage. Although quit lines are effective, the absolute quit rates achieved are modest[13-14], and this is particularly true among low-income callers[15-16].

The current project seeks to evaluate ways to improve outcomes for low-education, uninsured or Medicaid-eligible smokers who do not quit with standard quit line treatment. The current proposal will use an efficient factorial design to evaluate four evidence-based strategies to improve follow-up WTQL care offered to socioeconomically disadvantaged callers who report smoking four months following initial WTQL treatment comprising a single counseling call and 2-week supply of a single nicotine replacement therapy. These strategies include: increasing the intensity of nicotine replacement medication (among those medically cleared to use such medications), increasing the intensity of WTQL counseling, helping callers enroll in an evidence-based smoking cessation texting support program (SmokefreeTXT), and offering moderate financial incentives for engagement in counseling and SmokefreeTXT. This evaluation will identify which program enhancements, and which combination of program enhancements, improve abstinence rates in this high-priority and high-risk segment of the population served by the WTQL.

II. GOALS/AIMS

The overarching aim of the proposal is to evaluate ways to enhance quit line treatment for low-income smokers in terms of abstinence, costs, and reach. The specific aims of the project are listed below.

1. To assess the main and interactive effects of the four experimental factors (see below) on biochemically confirmed, 7-day point-prevalence abstinence at 6-month follow-up among low-education, uninsured, or Medicaid-eligible smokers who relapse following a standard WTQL intervention. Secondary abstinence outcomes are self-reported point-prevalence abstinence 12-weeks post-quit and continuous abstinence between months 1 and 6 post-quit.

Factors	***Levels***
1. Quit line counseling intensity	1. 1 vs. 4 proactive counseling calls
2. Nicotine replacement therapy (NRT) intensity	2. Patch (2 wks) vs. Patch+ Lozenge (4 wks)
3. SmokefreeTXT text messages	3. Proactive enrollment vs. none
4. Financial incentives for treatment engagement	4. $30-$150 vs. none

2. To evaluate the cost-effectiveness of each intervention component overall and in the context of the other components (i.e., additive and interactive effects and their impact on cost-effectiveness). The aim is to develop data to inform future quit line care planning and policy.

3. To identify demographic and smoking history moderators of treatment engagement and response and mediators of intervention effects (e.g., treatment engagement) on 6-month abstinence. This aim will identify sub-populations that may not benefit equally from enhanced WTQL services and will suggest ways to refine the treatments to enhance their efficacy.

III. METHODS

This program will evaluate services delivered by the WTQL (operated by Optum based in Seattle, WA). Evaluation data will be collected by the WTQL, but also from SmokefreeTXT, a public program sponsored by the National Cancer Institute and operated by ICF International based in Fairfax, VA. Follow-up biochemical verification of abstinence will be conducted in some or all participants claiming abstinence from smoking, either by mail or e-mail or by staff at the University of Wisconsin Center for Tobacco Research and Intervention (UW-CTRI) at one of several sites within driving range of UW-CTRI staff, as the WTQL does not have a physical presence in Wisconsin and therefore does not have the capability to collect these important data. UW-CTRI staff will also coordinate the evaluation effort and conduct evaluation analyses (with data from the WTQL and SmokefreeTXT and follow-up data collected by UW-CTRI).

A. Recruitment, inclusion and exclusion

Up to 1600 enrolled study participants will be Wisconsin residents who smoke and who completed the standard WTQL treatment 2.5-18 months prior to study recruitment. Participants will be recruited through the WTQL during a 2.5-18-month post-enrollment follow-up conducted by WTQL personnel. Callers to the WTQL are advised about this follow-up call at the time of enrollment as part of routine WTQL practice. At the time of enrollment in standard WTQL treatment, we will include a study flyer insert in the standard WTQL welcome mailing to all registered tobacco users (excluding those under 18 years of age and pregnant). The insert will inform participants that the WTQL may call them again in 4 months to see how they are doing and that, at that time, the participant may be able to get more support and earn money by joining a research study. The flyer encourages participants to stay in touch with the QL.

Recruitment will occur through proactive follow-up calls initiated by WTQL staff, supplemented by letters alerting prospective participants to the opportunity to receive additional services and to possibly join a paying research study evaluating ways to enhance quitline services. Only individuals who indicated that they were Medicaid-eligible, uninsured, or had a high school education or less at the time of enrollment, who completed a WTQL coaching call, and who meet the prerequisites described below will be contacted for follow-up. We anticipate that the WTQL will attempt to reach roughly 8,000-10,000 WTQL uninsured or Medicaid-eligible adult callers from target counties and screen roughly 3,000-4,000 of these to achieve our target sample of 1,408-1600 randomized enrollees over 3-4 years.

Prerequisites for study screening, as reported at time of WTQL registration:

1. Uninsured,covered by BadgerCareWisconsin or another Medicaid program, or has no more than a high school education
2. Live in Wisconsin
3. Age 18 or older
4. English speaking (<1% of WTQL callers request services in a language other than English)
5. Providing a unique telephone number not already registered in the WTQL database for this study (to prevent duplicate enrollment and to ensure that only one person per household enrolls)
6. Engaged in WTQL counseling

Inclusion criteria assessed at study enrollment are as follows (see uploaded Eligibility Screen):
1. Reported smoking at least 6 of the last 7 days at the WTQL follow-up enrollment call, with at least 5 cigarettes smoked on 1 or more days
2. Willing to set a new quit day within 2 weeks

Exclusion criteria are as follows:
1. Pregnancy, breastfeeding, or planning to become pregnant in the next 6 months at the time of study enrollment

B. Consent

Participants who meet inclusion criteria and who do not endorse exclusion criteria will be read a consent script by a WTQL staff member (see uploaded consent script). The consent script read to participants over the phone conveys the key points regarding study procedures; risks; and participant rights, protections, and benefits in accessible, lay language. Prospective participants will be given an opportunity to ask questions and raise concerns during this process, and these will be addressed prior to assessing participant consent to participate. Informed consent will be formally documented electronically in a WTQL database.

In late summer or early fall 2020, the Wisconsin Tobacco QuitLine will adopt 2 new targeted services. The first program is the American Indian Commercial Tobacco Cessation program that offers 12 weeks of combination NRT and 7 proactive calls from specially trained Quit Coaches. This is far more intensive than the standard QuitLine program, and more intensive than services offered in the research study. The second program is a year-long Text2Quit program, a text-message-based program similar to the SmokefreeTXT program to which half of study participants will be randomized in this study. Prospective participants will be advised that Text2Quit will not be offered in the study, and that only half of participants will be randomized to SmokefreeTXT. Once these programs have been implemented at the Wisconsin Tobacco QuitLine, prospective participants will be informed about the availability of these services for American Indian/Alaskan Native or 18-24 year olds and told how these differ from the cessation services in the research study prior to being asked if they want to consent. This information will also be noted in the written study information sheet mailed to participants after enrollment over the phone.

Following enrollment for those who consent, WTQL staff will send out a letter in hard copy to the participant at the address provided. This letter will remind the participant of study procedures and will include a detailed study information sheet containing all required elements of informed consent and HIPAA authorization.

The WTQL currently obtains consent for standard treatment over the phone and, given the remote nature of the interventions the WTQL delivers, an oral consent process for the evaluation study is the most in keeping with current WTQL procedures and is most likely to protect the validity of the

evaluation study. Mailing a written consent form and waiting for this to be returned would result in an evaluation study sample that is less representative of the target population (i.e., selecting for those most motivated or most stably motivated, those with the most reliable mail service, those will the highest literacy, those with the highest level of trust in counseling, etc.). Introducing a delay between enrollment and treatment delivery for the sole purpose of documenting written informed consent would undercut the knowledge to be gained by the study without substantially enhancing subject protections. Requiring written informed consent and HIPAA authorization would reduce the real-world relevance of this study designed to evaluate enhancements of a public, real-world telephone-delivered smoking cessation support service. For these reasons, we propose to use an altered HIPAA authorization because it would be impracticable to obtain authorization with all elements required under the HIPAA Privacy Rule to conduct our research study. We also seek to waive some of the required elements of consent and HIPAA authorization over the phone in to order to reduce participant burden and to enhance comprehension of the material read to participants over the phone, without omitting any key information pertinent to risks, rights, or protections. The mailed study information sheet that will follow the telephone enrollment and oral consent process will include all required elements of consent and HIPAA authorization (including a statement regarding how long permission to use the PHI will last, how to withdraw permission for PHI use, and the mandatory statement that the PHI could be redisclosed without subjects' permission in the consent/authorization and enrollment process). The study information sheet also details how to withdraw from the study and revoke authorization to use PHI, without penalty or punishment. We believe the use of the PHI in this study presents minimal risk to subjects and that we are using the minimum necessary PHI to conduct this study.

Other considerations also support the use of oral consent to participate in this evaluation study and to have patient data used for the purposes of program evaluation. Without patient level data, the study would not be able to analyze any person factors related to the success of smoking cessation attempts (Aim 3). The level of risk involved is also low, and the study will evaluate ways to enhance WTQL effectiveness using minimal risk interventions (a counseling intensity currently available to millions of Americans who smoke, FDA-approved, over-the-counter medications available nationwide, a cessation-focused texting program in which 5,000 Americans enroll monthly, and moderate incentives unlikely to be coercive). In addition, the data to be collected are not highly sensitive (i.e., we do not ask about illegal behaviors) and will be securely transmitted and stored.

With regard to compensation and incentives, the consent script and mailed study information sheet will make clear distinctions between compensation for completing study follow-up assessments (available in all conditions), and incentives for treatment engagement (available in varying amounts to those in only half of conditions). This is important for the sake of transparency, but also to prevent attrition among those randomized to conditions that offer no or minimal incentives.

C. Baseline assessment

Following oral consent, the WTQL staffer will: conduct a brief baseline assessment to supplement the smoking, quitting, and health history captured routinely at WTQL registration (see uploaded WTQL Registration Data); help the participant select a target quit-date within 14 days; and collect information on 1-3 contacts who could help the WTQL get in touch with participants who move or experience phone interruptions (with participant consent).

D. Randomization

Randomization will occur immediately after baseline data are collected after oral consent is obtained and will be conducted by WTQL staff using randomization tables developed at UW-CTRI. Randomization will be blocked by ethnicity and race to ensure an even distribution of participants of these factors that may relate to intervention success. Enrollees will be randomized to one of 16 conditions, as shown above. No placebo will be used in this real-world trial. Every enrollee will receive no less than the current standard in WTQL care, a single counseling session, a 2-week supply of a single nicotine replacement therapy (if medically eligible for nicotine medication), and unlimited access to a WTQL Quit Coach by request.

Following randomization, WTQL staffers will describe the randomized interventions in accessible language. This will include description of the counseling offered; description of the medications to be mailed to the participant; information about SmokefreeTXT; and a description of relevant incentives and their contingencies (i.e., $30 for each counseling call lasting, $30 for staying in SmokefreeTXT for the full 6 weeks post-quit).

E. Welcome mailing

Following the enrollment call, WTQL staff will mail enrollees a welcome package containing the following:

1. A welcome letter with incentive schedule, treatment information, and WTQL and UW-CTRI contact information for questions, concerns, or additional Quit Coaching (see uploaded Welcome Letter)
2. A copy of the study information sheet containing all the information agreed to over the phone (see uploaded Study Information Sheet)
3. SmokefreeTXT description and copies of current program enrollment privacy policy and terms of use (if in SmokefreeTXT condition and agrees to receive information about SmokefreeTXT, see uploaded SmokefreeTXT information)

Study medications will be send in a separate package with medication instructions, if applicable (content depends on condition, but materials will be standard WTQL materials for each agent, patch and lozenge, see uploaded medication instructions), in accordance with standard WTQL practice (a vendor dispenses medication directly to enrollees on behalf of the WTQL).

F. Study treatments and experimental conditions

All study participants will be offered a second round of standard evidence-based WTQL treatment comprising a single counseling call and, if medically eligible, a 2-week supply of a single nicotine replacement therapy (this will be the nicotine patch for everyone to reduce heterogeneity in regimens and because data suggest that adherence to nicotine patch therapy is superior to that of nicotine gum or lozenge). Everyone will receive care that meets or exceeds the current standard of care for WTQL services to Wisconsin residents. Participants will be randomized to additional interventions to supplement this base treatment. None of the interventions to be added as supplements to standard WTQL is experimental, as all have data to support their efficacy. In fact, a multi-call protocol used to be the standard of care for the WTQL prior to budget cuts. Adding a fast-acting nicotine replacement therapy (NRT) to nicotine patch (called combination NRT) is an accepted treatment supported by meta-analyses[17-19] and approved by FDA. Meta-analyses also support the effectiveness of text messaging support programs for smoking cessation like SmokefreeTXT[20-21]. Growing evidence, including two previous program evaluation projects for Medicaid-eligible Wisconsin smokers coordinated by UW-CTRI, supports the efficacy of incentives as a driver of treatment engagement and, thereby, of long-term abstinence rates[22-23]. The

Cell	WTQL	NRT (if eligible)	Texts	Incentives
1.	1 call	2 wk Patch	SmokefreeTXT	Yes, up to $60
2.	1 call	2 wk Patch	SmokefreeTXT	No
3.	1 call	2 wk Patch	None	Yes, up to $30
4.	1 call	2 wk Patch	None	No
5.	1 call	4 wk Patch+Lozenge	SmokefreeTXT	Yes, up to $60
6.	1 call	4 wk Patch+Lozenge	SmokefreeTXT	No
7.	1 call	4 wk Patch+Lozenge	None	Yes, up to $30
8.	1 call	4 wk Patch+Lozenge	None	No
9.	4 calls	2 wk Patch	SmokefreeTXT	Yes, up to $150
10.	4 calls	2 wk Patch	SmokefreeTXT	No
11.	4 calls	2 wk Patch	None	Yes, up to $120
12.	4 calls	2 wk Patch	None	No
13.	4 calls	4 wk Patch+Lozenge	SmokefreeTXT	Yes, up to $150
14.	4 calls	4 wk Patch+Lozenge	SmokefreeTXT	No
15.	4 calls	4 wk Patch+Lozenge	None	Yes, up to $120
16.	4 calls	4 wk Patch+Lozenge	None	No

proposed evaluation is important to see how well these interventions work among low-education, uninsured, and Medicaid-eligible smokers who did not quit with standard treatment (overall and in combination with one another), how cost-effective they are, how consistently they help subpopulations of smokers, and how they can best be combined to optimize their effects.

G. Detailed study procedures: recruitment and retention

Personnel from three organizations will have contact with study participants or their data. Staff at Optum, operator of the WTQL, will recruit, screen, consent, assess, randomize, and deliver treatment to participants. Personnel from ICF International, the operator of SmokefreeTXT, will not have direct contact with participants, since SmokefreeTXT is a fully automated texting program. Personnel at ICF International will, however, transmit participant data on use of SmokefreeTXT, cell phone numbers, and basic demographics. Finally, UW-CTRI personnel will interact with participants to conduct follow-up telephone interviews (12- and 26-weeks post-quit

day) and to collect biochemical saliva samples via mail, collect breath carbon monoxide (CO) test results via email, or biochemically verify abstinence at brief in-person visits at UW-CTRI offices (26-weeks post-quit day) among some or all of the participants who claim they have not smoked in the last 7 days at the 26-week follow-up interview. The table below details the activities that participants will complete during the study and identifies the entity responsible for implementation. Coordination of all activities will be led by UW-CTRI and UW-CTRI will perform all data analyses.

As shown below, recruitment will be conducted by designated staff at the WTQL by inviting the subset of registered quit line callers meeting study eligibility prerequisites to screen for a study evaluating program enhancements for smokers who want to quit within 2 weeks. Staff at UW-CTRI will communicate with the WTQL team working on this project at least monthly to monitor progress in recruitment, treatment engagement (i.e., retention), and implementation challenges. To promote recruitment, WTQL staff will make at least 5 attempts to reach prospective enrollees. Similarly, to promote retention, WTQL Quit Coaches will make at least 5 attempts to schedule/deliver each counseling session by telephone. Letters will be sent to individuals meeting study prerequisites to encourage them to reconnect with the WTQL. Staff at the WTQL will be responsible for processing incentive payments to participants as soon as possible after they are earned. Staff at the WTQL will also be responsible for assessing adverse events related to its interventions in accordance with their existing protocols (i.e., staff physician review).

Optum will give participants randomly assigned to SmokefreeTXT who agree to receive information about SmokefreeTXT instructions about how to enroll. In turn, ICF International will transmit results data from SmokefreeTXT via secure FTP to help staff at Optum determine whether or not a participant has earned the $30 incentive for staying enrolled in SmokefreeTXT through 6 weeks post-quit day.

UW-CTRI will receive data on enrollees to facilitate follow-up assessments. These data will include a study identifier and contact information (name, telephone number, address) needed to conduct telephone interviews and to find the nearest UW-CTRI office for biochemical verification of claims of abstinence at the 26-week follow-up. UW-CTRI staff who conduct follow-up interviews and verification will be blind to study condition and will not have access to other participant data (e.g., smoking history) prior to the end of data collection. All data transmissions to and from UW-CTRI will occur via secure FTP.

Assessment Plan

1. <u>Baseline</u> assessment (See uploaded registration data from the WTQL). Specific fields collected at initial registration for WTQL services will be pulled for study use and analyses. These items include age; gender; race; ethnicity; county; education; insurance status (uninsured,in BadgerCare Plus/Medicaid, or privately insured (if low in education)); chronic health conditions; registration date; how heard about WTQL; and smoking history (multiple items. These data will be collected for all consented enrollees and patient-level data without direct identifiers will be collected for those who are unreachable, are ineligible, or decline to enroll.

2. <u>Enrollment</u> assessment (see uploaded Post Consent Baseline assessment). Following oral consent, additional data that are not routinely assessed at the standard post-WTQL enrollment follow-up will serve as baseline assessments for the evaluation study. Items include a detailed assessment of smoking in the past 7 days (how many cigarettes smoked on each day) and other current eligibility criteria. In addition, items will assess supplemental indicators of socioeconomic status, recent use of nicotine products and smoking cessation therapies and associated costs, use of healthcare over the past 6 months (for cost-effectiveness analyses), frequency of texting, indicators or relapse proneness, brief psychiatric

status/history screener (without questions on illicit substance use), and menthol cigarette use. The WTQL will also confirm registration data whenever someone re-enrolls in treatment, as per their standard protocol. The target quit date will also be collected.

3. Engagement assessment. Engagement will be tracked using data routinely collected in the course of existing services, including the number and duration of WTQL calls completed and use of the WTQL Text2Quit program (among 18-24 year olds who enroll in this program outside of their study enrollment call, which is possible), the duration of enrollment in SmokefreeTXT and the number and content (e.g., craving or mood ratings; smoking status reports; requests for help with craving, mood, or slips) of inbound, subject-generated messages to SmokefreeTXT. Medication use will be assessed via self-report at 12- and 26-week follow-up telephone interviews.

4. Follow-up assessment (see uploaded follow-up assessment). At 12- and 26-weeks following the target quit day, participants will be asked to report on recent use of tobacco and smoking cessation products and associated costs, treatment satisfaction, and levels of candidate mediators of intervention effects (nicotine withdrawal distress, confidence, motivation, exposure to smoking triggers, and perceptions of stress, support, or control). Participants will earn $30 for completing each of these calls. At the 12-week call, participants will also be asked whether the research team may email them reminders about the final follow-up at 26 weeks.

   At 26 weeks following the target quit day, individuals claim to have not had even a puff of a cigarette in the past 7 days may be asked to schedule a verification visit at which they will be asked to provide a breath sample for carbon monoxide (CO) testing using the commercially available Micro+Smokerlyzer® from Bedfont Scientific Ltd. and a urine sample for cotinine testing to confirm abstinence. These biochemical verification procedures present minimal risk, as they require only that participants hold their breath for 15 seconds before blowing into a CO detector and that participants urinate in a cup. In order to achieve a high level of abstinence verification, and in light of participant travel involved, participants will receive $40 for completing this visit. Participants may be asked to complete alternative testing to verify abstinence remotely. This may entail mailing back a saliva sample for cotinine testing or emailing the research team a CO test using a personal CO monitor, and will similarly earn participants $40.

   We have defined the window for the follow-up periods broadly (11-18 weeks for the follow-up targeted to 12 weeks post-quit and 25-38 weeks for the follow-up targeted to 26 weeks post-quit) in order to maximize retention in a participant population that may experience socioeconomic conditions that impede connection (e.g., phone disconnections, relocation). We seek to maximize follow-up ascertainment by keeping the follow-up windows broad and making many attempts to contact participants within these windows. We will leave discreet voicemails (identifying the study by its nickname, the IQS Study, without revealing the nature or purpose of the study) to participants who consented to voicemails at the time of enrollment in quitline services. Data analysis strategies will take into account the breadth of the follow-up windows and will use late follow-up data (e.g., those more than 2 weeks from the target follow-up date) to inform sensitivity analyses to assess the robustness of results under varying inclusion criteria for these cases and assumptions about missingness in a tighter follow-up window. We will cease attempts to complete a follow-up if a participant declines the interview, complains about our calling, or reaches the end of the follow-up window without being reached.

	Schedule of Study Activities	*Imple-menter*
Standard WTQL Treatment	• Registration (tobacco history, demographics, insurance, education, chronic health conditions, county,)<br>• 1 proactive counseling call<br>• 2-week NRT monotherapy starter kit<br>• Unlimited coaching calls (initiated by smoker)<br>*Anticipated N=7,963*	WTQL
~4-Month Follow-Up Eligibility Screening (Attach-ment A)	• Assess smoking status (past 7 days)<br>*Anticipated # Reached N=3,185*	WTQL
	• Screen relapsers for inclusion/exclusion criteria<br>• Set quit date within 2 weeks<br>*Anticipated # Eligible N=2,548*	WTQL
Oral Consent (uploaded)	• Describe study<br>• Elicit and answer questions<br>• Obtain & document consent<br>*Anticipated # Enrolled N=1,408-1,600*	WTQL
Collect Baseline Data (Attach-ment F)	• Socioeconomic indicators (income, employment)<br>• Cessation history<br>• Recent nicotine & e-cig product use and costs<br>• Past 6-mo healthcare use[25]<br>• Frequency of text messaging<br>• Psychiatric history[26]<br>• Perceived stress<br>• Menthol use<br>• Indicators of relapse proneness (e.g., exposure to others smoking, craving when abstinent)<br>• Use and costs of stop-smoking treatments since last QL call<br>• 1-3 collateral names & phone numbers	WTQL
Initiate Treatment	• Randomize to treatment<br>• Schedule first/only WTQL counseling call<br>• Describe medication regimen<br>• Describe assessment schedule<br>• Describe incentives schedule, if applicable<br>• Ask patients if want information about how to enroll in SmokefreeTXT, if applicable	WTQL
Welcome Mailing (Attach-ments B-D)	• Medication (sent in a separate package to those medically eligible)<br>• Medication instructions, if applicable<br>• Target quit date & follow-up reminders<br>• Contact information for WTQL & UW-CTRI (to reduce call rejection)<br>• Incentive reminders, if applicable<br>• SmokefreeTXT descripition, terms of use & privacy policy, if applicable<br>• Study information sheet containing all required elements of consent and HIPAA authorization	WTQL
12-Week Follow Up (Attach-ment G)	• Self-reported tobacco use & costs in past 7 days & since target quit date<br>• Treatment use, adherence, satisfaction, & costs<br>• Candidate mediators (perceived Quit Coach support; reduction in exposure to smoking/triggers; quitting motivation & confidence)	UW-CTRI
26-Week Follow Up (Attach-ment G)	• Self-reported tobacco use & costs in past 7 days & since target quit date<br>- Those claiming abstinence in last 7 days may be invited for CO and/or cotinine testing at a research office, or via mailed saliva collection kit or personal CO monitor for verification of abstinence, and will be paid $40 payment for test completion.<br>• Treatment use, adherence, satisfaction, & costs<br>• Mediators (reduction in exposure to smoking/triggers; motivation & confidence)	UW-CTRI

Data management, transmission and storage

The WTQL will collect data in a manner consistent with its current practices, with the exception that additional fields will be assessed at the WTQL follow-up for the first round of treatment (i.e., the call at which participants will be enrolled in this evaluation study). The WTQL will also record data on incentives earned and dispensed for those in the incentive condition. The WTQL maintains a secure, HIPAA-compliant database for its operations. This database will be modified for the current project to collect the additional data needed to achieve the study aims.

SmokefreeTXT also collects data on enrollee cell phone number, basic demographics, quit day, and engagement with the program (inbound messages from enrollees) in a secure and HIPAA-compliant manner. No additional data will be collected by SmokefreeTXT for the purposes of this evaluation study. SmokefreeTXT will securely transmit data on enrollee status (not reached, declined, dropped out prior to end of the program, or completed the program) at the end of the six-week program to the WTQL so staff there can process incentives for those eligible for the $30 incentive for completion of the SmokefreeTXT program.

Staff at UW-CTRI will receive data on participants due for follow-up from the WTQL (including names and contact information) on an ongoing basis (e.g., monthly) via secure FTP transfer. Because CTRI is the entity overseeing the WTQL contract for the State of Wisconsin and all data collected through its service to Wisconsin residents, this follows long-established, HIPAA compliant data transmission procedures.

After all participants in the study reach the end of the 26-week follow-up period:

- The WTQL will send UW-CTRI relevant registration, baseline, randomization, and treatment engagement data to UW-CTRI via secure FTP transfer. This will include data on enrollees, but also data on potentially eligible WTQL callers who did not complete the enrollment call or who did not meet eligibility screening at the enrollment call (to evaluate the representativeness of the enrolled sample). For participants who do not complete the full consenting process, UW-CTRI will obtain and keep data with no HIPAA identifiers other than an assigned WTQL ID#, which is used to verify what contract service was provided.

- ICF International will send data on SmokefreeTXT utilization at the end of the project. These data will include cell phone numbers and data regarding the volume and content of messages sent by participants to SmokefreeTXT (e.g., requests for help with cravings, negative moods, or slips; responses to SmokefreeTXT queries regarding craving, mood, and smoking) and the date of withdrawal from SmokefreeTXT (if relevant).

- At UW-CTRI, data from the WTQL and SmokefreeTXT will be linked to 12- and 26-week follow-up data. All collated study data will be stored in a HIPAA-compliant and secure manner on Department of Medicine servers with UW-CTRI-controlled password access. Staff trained in human subjects research and HIPAA compliance will link these datasets using a random subject identifier and will then de-identify the data for analyses.

Although this program will evaluate interventions delivered by the WTQL, the follow-up data collection procedures and collation of data from multiple sources/sites necessary for thorough program evaluation is the responsibility of the investigators in the study at UW-CTRI. To achieve the study aims, UW-CTRI investigators require access to identifiable patient data.

Treatment procedures description

The WTQL offers a standard, evidence-based protocol comprising a single 20-minute counseling call (plus *ad hoc* calls as initiated by the participant) and a two-week supply of an over-the-counter nicotine replacement therapy. This will be the treatment that all study enrollees will receive in the 2.5-18 months preceding study enrollment and will be the base treatment in the second round of intervention to be evaluated in terms of reach, efficacy, and cost-effectiveness in the proposed study. Four interventions will be evaluated in a fully crossed design:

- Augmented counseling (4 proactive, scheduled telephone counseling sessions with a quit coach rather than the usual-care 1 call). The content of this counseling will follow existing, evidence-based protocols used by the WTQL in states that contract for this more intensive service.

- Augmented nicotine replacement therapy (4 weeks of nicotine patch plus nicotine mini-lozenge rather than the usual-care two-week supply of nicotine patch therapy), for those meeting WTQL NRT eligibility criteria. These FDA-approved, over-the-counter medications will be dispensed with instructions to use that follow package insert specifications (e.g., using lower, 2-mg dose mini-lozenges for individuals who do not smoke within 30 minutes of waking; dispensing lower, 14-mg dose patches to individuals who smoke fewer than 10 cigarettes per day), in accordance with current WTQL practice and documentation.

- Adjunctive SmokefreeTXT support (proactive enrollment in this 6-8-week text message-based cessation support program versus none). This national program is offered at no cost to users (apart from data/minute costs charged by cellular providers about which enrollees are advised at enrollment) and is sponsored by the National Cancer Institute. This program sends up to 5 messages per day for up to 2 weeks prior to the target quit day and 6 weeks following the target quit day (total duration may be longer if participants elect to change the target quit day). The text messages offer tips to help enrollees achieve their goal of quitting smoking and offer real-time interactive tips when participants text in responses indicating cravings, negative moods, or slips. Participants have the option to reset quit dates and have the option to unsubscribe at any time by texting STOP to the program.

- Incentives for engagement ($30 for each counseling sessions and/or staying enrolled in SmokefreeTXT for six weeks versus none). These incentives are designed to bolster motivation to stay engaged in treatment and to offset the costs and effort of treatment engagement (in terms of cell phone minutes). Research suggests that incentives improve treatment engagement which, in turn, increases success in quitting smoking[27].

Analysis Plan

To assess Aim 1, we will test the main and interactive effects of counseling intensity, NRT intensity, SmokefreeTXT, and engagement incentives on biochemically confirmed, 7-day point-prevalence abstinence at 6-month follow-up. Secondary abstinence outcomes are self-reported point-prevalence abstinence 12-weeks post-quit and continuous abstinence between months 1 and 6 post-quit.

Measures, Populations and Analysis:
Data for the primary, abstinence-focused analyses will include randomized levels of the treatment factors and their interactions as independent variables, binary abstinence outcomes based on self-reported abstinence (at 12-week follow-up) and biochemical verified abstinence (at 26-week follow-up only) as the dependent variables, and covariates such as demographics and smoking history collected at registration or enrollment.

The population will be the eligible, consented, enrolled subjects who were randomized to treatment. Participants who were randomized but did not complete the study will be included in analyses (unless they requested that their data not be used upon withdrawal from the study). Analyses will occur in the full intent-to-treat sample.

Logistic regression analyses will be used to estimate intervention main and interactive effects on binary abstinence outcomes. Both unadjusted models and models adjusted for covariates will be run. Separate models will be run for different outcomes (e.g., biochemically confirmed abstinence at 26-weeks post-quit, self-reported abstinence at 12 weeks post-quit, continuous abstinence between 4 and 26-weeks post-quit) under different assumptions regarding missing abstinence data (e.g., assuming all missing are smoking, assuming 80% of missing are smoking, using multiple imputation).

Data Sources and Collection: The primary outcome data will be collected by UW-CTRI via telephone follow-up interview and in-person, mailed, or emailed validation of abstinence via CO breath testing and/or urinary or salivary cotinine testing. Covariates will be assessed by WTQL staffers at registration or enrollment and transferred securely to UW-CTRI for analysis.

To assess Aim 2, we will evaluate the costs and cost-effectiveness of each intervention component overall and in the context of the other components (i.e., additive and interactive effects and their impact on cost-effectiveness).

Measures, Populations and Analysis:
Cost data will come from: research participants who will report on their out-of-pocket costs of tobacco and treatment and health care utilization at enrollment and follow-up; Optum who will report on intervention delivery, supply, and support costs; SmokefreeTXT who will report minute/data use costs associated with cumulative message volume and the estimated cost per user for the program; and from public data sources (on the costs and savings associated with cessation and continued smoking).

The population will be the eligible, consented, enrolled subjects who were randomized to treatment. Participants who were randomized but did not complete the study will be included. Analyses will occur in the full intent-to-treat sample whenever possible, using model estimation that permits missing data. Utilization rates have important implications for intervention costs, so both low and high utilizers of intervention components will contribute important data to these analyses.

Cost and efficacy data (abstinence outcomes assessed in Aim 1) will be used to estimate the costs per verified quit by treatment condition, in accordance with recommendations for cost-effectiveness analyses[28]. These data will also be used to estimate costs per quality-adjusted-life-year (QALY) saved, based on prior estimates of QALY associated with smoking status. As recommended by Feirman et al. [29] sensitivity analyses will be conducted, using alternative QALY measures to assess robustness of estimates. We can

use data from the project's factorial design to generate smoothed estimates of the costs per quit and costs per QALY for different combinations, using multivariate regression analysis. We will also estimate potential savings related to reduced smoking levels, as a function of previous smoking frequency and the estimated effects of the interventions on quitting or self-reported post-intervention smoking frequency. This approach could use information from the factorial design to create smoothed estimates of personal savings.

Data Sources and Collection: Participants will self-report health care utilization and spending on tobacco and cessation treatments; Optum will report on WTQL intervention costs; and SmokefreeTXT will report on the volume of text message exchanges and total costs per user. UW-CTRI will collate data from these sources for analyses.

To assess Aim 3, we will identify demographic and smoking history moderators of treatment engagement and response and mediators of intervention effects (e.g., treatment engagement) on 6-month abstinence. Such analyses examining treatment by moderator interactions or examining treatment effects in subgroups of participants (e.g., the uninsured versus Medicaid-eligible; men versus women) will identify sub-populations that may not benefit equally from enhanced WTQL services using data collected by the WTQL at registration or study enrollment. We will also explore the extent to which treatment engagement (measured throughout counseling and SmokefreeTXT and at follow-up for medication) and cessation motivation and confidence (assessed at follow-up) differed across treatment conditions, and the extent to which such differences may mediate or account for treatment effects on abstinence.

Measures, Populations and Analysis:
Self-report demographic, smoking history, health status, psychiatric history, and texting use collected at WTQL registration or study enrollment will be examined as candidate moderators of treatment response. Mediators (treatment engagement; perceived support; reduction in exposure to smoking/triggers; quitting motivation and confidence) will be assessed during treatment (in the case of treatment engagement) or during follow-up telephone interviews at 12- and 26-weeks post-quit.

The population will be the eligible, consented, enrolled subjects who were randomized to treatment. Participants who were randomized but did not complete the study will be included (unless they requested that their data not be used upon withdrawal from the study). Analyses will occur in the full intent-to-treat sample whenever possible, using model estimation that permits missing data.

Evaluation of moderators will be accomplished with moderated logistic regression models that include effect-coded intervention main and interaction effects on abstinence, a given moderator, and interactions of the moderator with the intervention effects. Mediation analyses will include linear regression models in which mediators are regressed on intervention main effects and interactions, logistic regression models in which binary abstinence outcomes are regressed on both intervention variables and mediators, and estimates of the mediated effect. Formal mediation analysis computed via the SAS PROCESS macro[30].

Data Sources and Collection: Candidate moderators will be assessed via participant self-report by the WTQL during initial registration or study enrollment. Treatment engagement mediators will be assessed by the WTQL (for counseling completion), SmokefreeTXT (for SmokefreeTXT completion and interactions), and by UW-CTRI at follow-up telephone interviews (for self-reported patch and mini-lozenge use). Other candidate mediators

(cessation motivation and confidence) will be assessed by UW-CTRI during 12- and 26-week telephone follow-up interviews.

Products in the Evaluation/Research Reports: We anticipate writing at least four different research articles for publication in peer-reviewed journals based on this project. Aim 1 results regarding intervention efficacy will be published in a single manuscript. Aim 2 results regarding costs and cost-effectiveness will be published in a separate manuscript. Aim 3 results on moderators of intervention effects will be published in a third manuscript and mediation results will be published in a fourth manuscript. It is also possible that we will publish a separate protocol and process description of the experimental study while the study is underway.

H. Human Subjects Protections and Data Sharing

Describe any potential direct benefits to subjects. If there are no direct benefits, state this.

The health benefits of quitting smoking are considerable and the current study may increase the likelihood that study participants will successfully quit by proactively offering a second round of evidence-based treatment to WTQL callers who did not achieve lasting abstinence following a prior round of treatment. All treatments used in this study have been demonstrated to be effective compared to unaided quitting in clinical practice guidelines and/or multiple research studies.

Describe the potential benefits of this research to society.

This experiment will generate knowledge regarding the efficacy, cost-effectiveness, and reach of interventions that can be delivered remotely and inexpensively (with few physical or financial barriers) to a high-priority population, low-income smokers who have benefited less from existing interventions and delivery methods than have higher-income smokers. The results of this study have the potential to identify highly efficient and cost-effective means of enhancing abstinence rates in this high priority population, and thereby to reduce socioeconomic disparities in the burden of tobacco use. These results also have the potential to inform the design and delivery of public benefit programs (e.g., state quit lines) serving the target population of low-income smokers. This may reduce the substantial individual and societal costs of tobacco use and related productivity losses, disease, and death.

Describe any potential psychosocial risks to subjects, such as psychological stress or confidentiality risks (including risk to reputation, economic risks, and legal risks).

Risks associated with this research are judged to be minimal, consistent with the minimal risks associated with use of nationally available smoking cessation quit line and text messaging services.

The processes of collecting treatment engagement, self-report, and biochemical (breath and urine) data carry minimal risks, and sensitive questions (e.g., regarding illegal behaviors) will not be asked. Despite this and the safeguards to protect participant information we will apply, breach of confidentiality is a risk that cannot be eliminated completely. There is a remote possibility that a subject's identity will become known to unauthorized persons.

In addition, NRT may cause side effects. NRT risks are well-documented, given their wide-scale use and over-the-counter availability (in both of the forms to be used here: patch and lozenge). Use of the nicotine patch may lead to dizziness, nausea, vomiting, rapid heartbeat, weakness, skin irritation at the site of application, sleep disturbance, rapid heartbeat, headache, and muscle

or joint aches and stiffness. According to the package insert, the most likely side effects associated with the nicotine lozenge are heartburn, hiccup, nausea, upper respiratory tract infections, coughing and sore throat. These NRT side effects are typically mild to moderate, even when individuals smoke while using nicotine replacement[31-33] and the symptoms typically subside when NRT use is reduced or discontinued. It is important to note that quit lines currently screen clients for medication eligibility, dispense NRT via mail in many states, including Wisconsin, and that some currently dispense combination nicotine replacement (patch with gum, or patch with lozenge). As such, the proposed study procedures should not pose additional risks of medication side effects above and beyond current quit line practices.

An additional risk is the experience of aversive nicotine withdrawal symptoms upon cessation, such as sleep disturbance, hunger, craving, difficulty concentrating, and negative mood. These symptoms are temporary and pose minimal health risk.

Providing breath and urine samples for biochemical verification of abstinence carries minimal risks, as these procedures are non-invasive. Risks from study participation are deemed to be much lower than the known health risks of continued smoking.

Describe how ALL the risks of the study will be minimized.

To protect against breach of confidentiality or violations of privacy, we will ensure that data are stored on secure, encrypted servers at all participating sites, and that transfer of data between sites is conducted via HIPAA-compliant means. A unique study ID will be used to store all data on individual participants and information linking that study ID to participant identifying information will be maintained by the data manager. Data being used for analysis will be identified with the study ID only. Participant contact information will only be available to study staff having direct contact with participants. Data will be stripped of direct identifiers (limited datasets) or completely de-identified prior to sharing with others outside the IRB-approved research team.

To protect against adverse reactions to over-the-counter nicotine medication, the WTQL will screen participants for eligibility using their standard, physician-reviewed protocol over the phone and participants will receive simple, clear instructions in their proper use in the mailing sent post-enrollment. These forms will advise participants of common side effects and will describe ways to prevent or address these (i.e., putting a new patch on a different part of the body each day, taking the patch off at night if sleep disturbance is too bothersome). These medication instruction forms and all written study materials will be written at no higher than a sixth-grade reading level. In addition, people who report recent cardiac-related surgeries or hospitalizations, and those with allergies or past negative reactions to nicotine replacement, will be screened out of medication eligibility at enrollment to further reduce risks of nicotine administration. All Quit Coaches receive training on smoking cessation pharmacotherapy and assess medical conditions that may preclude or complicate NRT use at registration. In addition, Quit Coaches have access to Optum physicians who can address questions or concerns about pharmacotherapies. Optum will conduct routine monitoring for adverse or unanticipated events according to their standing protocols.

Participants will be alerted to possible withdrawal effects at enrollment, will be offered over-the-counter medication to manage these symptoms (if medically eligible), and will be advised that these symptoms are typically mild and temporary.

Optum, the WTQL vendor, employs physicians to review medication protocols and has a rigorous adverse event monitoring protocol. All medications will be dispensed in ways that are consistent with the protocol and packaging instructions, with clear instructions. No more than a 4-week supply will be provided. Participants experiencing side effects will be advised to seek local

medical care as needed, with the understanding that the costs of the medical care will be the responsibility of the participant (or the participant's insurer), as discussed in the consent process.

The risk of aversive withdrawal symptoms will be addressed in two ways. First, standard WTQL practice addresses the nicotine withdrawal syndrome in an individually tailored manner. This will not change in the current study. Second, we will provide nicotine replacement to all participants, in accordance with best practice and current practices at the WTQL. All participants will be advised that they can call the WTQL for additional coaching calls, as needed, so even those in the single-scheduled-counseling-call-condition will have access to ongoing support regarding the management of nicotine withdrawal. Everyone medically eligible for NRT will receive at least nicotine patches (and half with receive lozenges in addition to patches) to address withdrawal during the first 2-4 weeks of the smoking cessation effort.

Explain why the risks to the subjects are reasonable in relation to the anticipated benefits.

This study carries minimal risk. None of the interventions to be deployed poses significant risk above and beyond standard WTQL treatment and every participant will receive at least one form of evidence-based smoking cessation treatment. No information about illegal behavior will be entered in databases. The risk of someone's identity becoming known to unauthorized persons is low. This risk is outweighed by the benefits of quitting smoking for any individual, and the benefit to society of having fewer people use tobacco products. This research has the potential to benefit low-income smokers in the future by helping to identify optimal combinations of remotely delivered interventions for population-wide dissemination. The proposed project will accelerate refinement of treatments with broad reach and low barriers to access using an efficient factorial research design. The risks associated with the use of over-the-counter nicotine patches and nicotine lozenges are minor relative to the risks of continued smoking, particularly with the medication eligibility screening used by the WTQL. If successful, this treatment approach could be used more broadly to reduce the considerable financial and personal costs associated with smoking-related disease. Therefore, the potential gain in knowledge regarding treatments and mechanisms of change associated with the proposed study outweigh the risks of participation.

Describe the provisions in place to identify and address unanticipated problems or complications.

The Principal Investigators (PIs) will be responsible for routinely monitoring study progress. Optum, the WTQL vendor, has an adverse event monitoring protocol and team in place and will alert the study PIs to adverse events among study participants. Any data safety concerns will be reported to the study PIs immediately and addressed. We will report any unanticipated problems to the IRB according to posted guidelines. A full data safety and monitoring plan is included at the end of this protocol.

Describe the precautions that will be used to ensure subject *privacy* is protected

Collection of sensitive information about subjects will be limited to the amount necessary to achieve the aims of the research. Participants will not be asked to disclose any information about illegal behavior.

Describe the measures that will be implemented by your research team to safeguard the identifiable subject information from unauthorized use or disclosure for both paper and electronic forms of information. Include how and where data will be stored.

Participant study data will be collected by the WTQL through a Computer Assisted Telephonic Interview (CATI) and will be stored on secure, password protected servers. Data will be

accessible only to assigned study staff for their study function; computer workstations will be password-protected, thus secured from unauthorized use. WTQL will transmit study data—enrollment information, randomization data, all assessment data, service utilization data, and identifying information to CTRI via a secure FTP site. On a monthly basis, identifiable research data including contact data needed to conduct follow-up interviews at UW-CTRI, will be transmitted through a secure FTP site by the WTQL to UW-CTRI.

Data received from participants in SmokefreeTXT are overseen by the site sponsor (National Cancer Institute). SmokefreeTXT data regarding completion of the program will be transmitted to the WTQL so staff there may process the $30 incentive for completing the program for those in the active incentive conditions. At the end of the study, more complete data on participant engagement in SmokefreeTXT will be transmitted to UW-CTRI for analyses via a secure FTP site.

Required patient identifying data will be stored in a separate file linked to all study data via a study identifier after it is received from WTQL and SmokefreeTXT. Confidentiality of participant data and information will be accomplished by using participant numbers as unique identifiers, allowing us to keep participant data separate from identifying information. Once transferred to UW-CTRI, data generated through study participation and data obtained on medical and psychiatric history from participants will be stored in secure databases under protections and procedures consistent with the guidelines and regulations of the UW School of Medicine and Public Health (UW-SMPH). Outside access is available only via an encrypted connection to the Department of Medicine Citrix server located at the UW Clinical Science Center in Madison. The servers at the UW-CTRI Madison office are physically secured in a locked room. Data backups are created nightly and stored in a locked safe. Significant safeguards have been implemented to protect data including virus and adware protection, firewall, access controls and encryption when appropriate such as wireless and remote access. All UW-CTRI staff members have completed HIPAA and human subjects training and are aware of the sensitivity of study-related data. The UW SMPH has developed school-wide data security policies and procedures. UW-CTRI data security policies and procedures conform to those of the SMPH. UW-CTRI will use an enterprise-level database that supports audit trails such as access, change logging, and more sophisticated access control for managing and tracking user access privileges. No publications or presentations resulting from this research program will contain any identifying information about individual participants. Following cleaning and verification of data at the conclusion of the study, all research data will be placed in a de-identified data set at UW-CTRI.

Data Safety and Monitoring Plan

The Data Safety and Monitoring Plan (DSMP) for this research comprises not only the research conducted directly by the University of Wisconsin Center for Tobacco Research and Intervention (UW-CTRI) researchers, but also research conducted by other investigators collaborating with UW-CTRI-funded projects. All investigators must agree to comply with the procedures outlined in this DSMP. This DSMP does not reduce any investigator's obligation to comply with the requirements of the Institutional Review Board (IRB) at his/her home institution or the IRB of any collaborating organizations.

Monitoring the progress of trials and the safety of participants. The Principal Investigators (PIs) are responsible for routine monitoring of study progress. This includes scheduled meetings with study staff at least monthly; review of weekly reports from the Wisconsin Tobacco Quitline (WTQL) vendor, Optum, whose personnel will recruit, screen, enroll, counsel, and compensate participants for treatment engagement; and review of monthly reports of use of SmokefreeTXT. Weekly reviews of WTQL reports and follow-up discussions as needed to address any concerns with Optum are vital as the study procedures, database queries, and reports are being developed,

launched, and refined in the first year of the project. Following roll-out, monthly review meetings will continue to monitor progress and to consider up- or down-regulating the rate of recruitment from month to month. Data reviewed at these meetings will include the number and type of participants enrolled, the number and reasons for exclusions from enrollment, the number of counseling sessions completed, the number of SmokefreeTXT enrollees who opt-out, and the timeliness and accuracy of incentive payments to participants. Adverse or unanticipated events reported at WTQL counseling calls will be addressed using standard WTQL protocols to address medication side effects, withdrawal, or other concerns. The DSMC will review reports on adverse events reported to the WTQL. Adverse or unanticipated events (e.g., failure to receive an earned incentive) reported by participants directly to UW-CTRI through proactive participant calls (participants will receive this contact information at enrollment) will be reported to Optum to record and attempt to resolve the event, and will be reported to the IRB, FDA, or NCI, as appropriate based on the severity and protocol relevance of the event.

To facilitate participant safety, study participants must meet study inclusion and exclusion criteria. Once enrolled, WTQL protocols will assess NRT eligibility and the presence of AEs and SAEs during treatment. Should either excessive risk to study participants and/or convincing evidence of lack of measurable benefit to study participants be determined, the study will be stopped and all participants notified in a manner appropriate to the nature of the risk and/or lack of benefit. When taking that step, the investigators will consult with the IRB and NCI.

Plans for assuring compliance with requirements regarding the reporting of adverse events. This DSMP requires that investigators notify NIH and the University of Wisconsin IRB in a timely manner (consistent with IRB and NIH policies) of the occurrence of any SAE or any AE which is severe, unexpected, and possibly related to study medication or protocol. Because this study involves pharmaceutical agents, if an unexpected SAE might be related to study drug use, both the Food and Drug Administration (FDA) and the manufacturer will also be notified within 5 days of investigators becoming aware of the event. Examples of unexpected SAEs would be untoward medical or intervention occurrences that result in death, are life-threatening, require hospitalization or prolonging of existing hospitalization, create persistent or significant disability/incapacity, or involve congenital abnormality/birth defects. Unanticipated AEs include less serious problems that merit reporting to the DSMC because they are severe, unexpected, and possibly related to study participation. Any SAE will be queried and reported even if it appears that the serious adverse event is unrelated to study participation. Because the interventions will be delivered by our WTQL partner, Optum, staff there will be responsible for assessing and managing any adverse events related to study medication in accordance with their existing protocols for monitoring NRT medication side effects or unanticipated health events during counseling calls. The study team will review Optum reports of unanticipated health events no less than bi-weekly, and Optum will notify the PIs of any severe, unanticipated, and possibly study-related adverse events in a timely manner so that NIH, FDA, and the IRB may be notified, as needed.

Adverse event assessment, recording, reporting, and investigation will be accomplished through staff training, structured/standardized assessments of untoward occurrences/events, and regular monitoring by Optum staff and the study team. The PIs have ultimate responsibility for ensuring that SAEs are detected and reported in a timely manner. Additionally, the IRB will receive an annual report of all SAEs and AEs meeting the criteria listed above.

Plans for assuring that any action resulting in a temporary or permanent suspension of an NIH-funded clinical trial is reported to the NIH grant program director responsible for the grant. The NIH grant program director will be notified within 5 days if the PIs deem it necessary to suspend

the clinical trial. In the case of a temporary suspension, the PIs will develop a plan for continuation of the study and discuss this plan with the NIH grant program director in a reasonable time frame.

Plans for assuring data accuracy and confidentiality and protocol compliance. The UW-CTRI PIs will develop protocols for assuring UW-CTRI data collection accuracy and protocol compliance (relevant to follow-up assessments). Such protocols will include data verification and protocol compliance checks. The UW-CTRI team will also work with Optum and SmokefreeTXT to ensure that their protocols have similar quality assurance standards and data security checks. The researchers at UW-CTRI will abide by the uploaded Data Use Agreement terms for use of SmokefreeTXT use data. The Data Manager and PIs will also be responsible for ensuring that the data for the project are securely transferred from Optum and SmokefreeTXT and stored at UW-CTRI, and that storage is in compliance with University and federal regulations and that no unauthorized persons have access (electronic or physical) to any participant-identifiable data. HIPAA regulations and guidelines are currently implemented at UW-CTRI and Optum, and all study staff must complete approved human subjects and HIPAA training programs.

Inter-site data transfers are accomplished via secure file transfer protocols (SFTP) using an internet server maintained by the UW School of Medicine and Public Health (UWSMPH) Department of Medicine. To protect the privacy of database records and the integrity of the network, this server is firewall-protected and is stored in a locked server room with a numeric keypad to restrict entry. The server is continuously scanned for the presence of viruses. A complete virus scan of all workstations also takes place once a week. Server system log files are scanned for unusual activity, which is immediately investigated. Network and Server Administration staff members apply critical and non-critical patches as needed. In addition, the UW-CTRI and the UWSMPH Department of Medicine also have multiple mechanisms for preserving confidentiality of research participants and providing data security in the transfer of data from participant machines to the SFTP server. The Department of Medicine web servers use Secure Socket Layer (SSL or https) technology to encrypt data exchanged between the client and the server. In addition, all online and offline components of the data systems described in the proposal will be accessible only through a login and password unique to each user. The security access levels for these login accounts are tiered and the features and privileges given to each staff member will be determined by the PIs and IT Manager. To further protect confidentiality, only the UW-CTRI IT Manager will be permitted to transmit data to the SFTP server. Finally, the Department of Medicine employs extensive data backup and server redundancy procedures and performs full backups to tape weekly of all servers, along with incremental and daily backups.

Data and Safety Monitoring Committee. In addition to the protections outlined in the DSMP (above), all research activities conforming to the NIH definition of a clinical trial will also have an independent Data Safety and Monitoring Committee (DSMC). This application includes a Phase IV clinical trial using FDA-approved medications. The DSMP specifies overall monitoring that will be conducted by Optum, as the intervention deliverer, and by the PIs, including timely reporting of AEs and SAEs. Every six months, the DSMC will convene to review the overall safety data, as well as data on safety summarized by treatment condition. As per NIH guidelines, the objective of these reviews will be to determine whether continued conduct of the trial poses any undue risk for participants.

The existing UW-CTRI DSMC is chaired by Dr. James Cleary, leader of the Cancer Control Program of the UW Comprehensive Cancer Center. Dr. Cleary is an experienced physician and clinical trial researcher with no involvement in any of this project's research activities. Dr. Cleary is joined on the DSMC by Dr. James Sosman and Dr. Burke Richmond. Dr. Sosman is Associate Professor of Medicine and Medical Director of the HIV/AIDS Comprehensive Care Program at UW Hospital and Clinics who has previously collaborated on a clinical trial of smoking cessation with

UW-CTRI. Dr. Richmond is an otolaryngologist who has served on independent DSMBCs for Phase II and III trials involving a nicotine vaccine. Neither has direct involvement with any of the proposed research. The PIs will report to the DSMC; the three DSMC members will be unblinded as to treatment conditions and will make the final determinations as to study continuation.

References

1. Frieden TR. The health consequences of smoking-50 years of progress. Executive summary. 2014. http://www.surgeongeneral.gov/library/reports/50-years-of-progress/exec-summary.pdf.
2. Clarke TC, Ward BW, Freeman G, Schiller JS. Early release of selected estimates based on data from the January–September 2015 National Health Interview Survey. 2016. http://www.cdc.gov/nchs/data/nhis/earlyrelease/earlyrelease201602.pdf. Accessed 31 May 2016.
3. Jamal A, Homa DM, O'Connor E, et al. Current cigarette smoking among adults - United States, 2005-2014. *MMWR Morb Mortal Wkly Rep.* 2015;64(44):1233-1240.
4. Centers for Disease Control and Prevention. Smoking rates for uninsured and adults on Medicaid more than twice those for adults with privated health insurance. 2015. http://www.cdc.gov/media/releases/2015/p1112-smoking-rates.html. Accessed 15 Sep 2016.
5. Clegg LX, Reichman ME, Miller BA, et al. Impact of socioeconomic status on cancer incidence and stage at diagnosis: selected findings from the surveillance, epidemiology, and end results: National Longitudinal Mortality Study. *Cancer Causes Control.* 2009;20(4):417-435.
6. U.S. Department of Health and Human Services. The health consequences of smoking: 50 years of progress. a report of the Surgeon General. 2014. http://www.surgeongeneral.gov/library/reports/50-years-of-progress/full-report.pdf.
7. David A, Esson K, Peruice A-M, Fitzpatrick C. Tobacco use: equity and social determinants. In: Blas E, Sivasankara Kurup A, eds. *Equity, social determinants and public health programmes*. Geneva, Switzerland: World Health Organization; 2010:199-218.
8. Centers for Disease Control and Prevention. Quitting smoking among adults - United States 2001-2010. *MMWR.* 2011;60(44):1513-1519.
9. Reid JL, Hammond D, Boudreau C, Fong GT, Siahpush M, Collaboration ITC. Socioeconomic disparities in quit intentions, quit attempts, and smoking abstinence among smokers in four western countries: findings from the International Tobacco Control Four Country Survey. *Nicotine Tob Res.* 2010;12 Suppl:S20-33.
10. Caleyachetty A, Lewis S, McNeill A, Leonardi-Bee J. Struggling to make ends meet: exploring pathways to understand why smokers in financial difficulties are less likely to quit successfully. *Eur J Public Health.* 2012;22 Suppl 1:41-48.
11. North American Quitline Consortium. Results from the 2015 NAQC Annual Survey of Quitlines. 2015. http://www.naquitline.org/?page=2015Survey. Accessed 13 Sep 2016.
12. Wisconsin Tobacco Quitline. Annual data report. 2015.
13. Borland R, Segan CJ. The potential of quitlines to increase smoking cessation. *Drug Alcohol Rev.* 2006;25(1):73-78.
14. Lichtenstein E, Zhu SH, Tedeschi GJ. Smoking cessation quitlines: an underrecognized intervention success story. *Am Psychol.* 2010;65(4):252-261.
15. Tzelepis F, Paul CL, Walsh RA, Wiggers J, Duncan SL, Knight J. Predictors of abstinence among smokers recruited actively to quitline support. *Addiction.* 2013;108(1):181-185.
16. Colorado Department of Public Health and Environment. Evaluation of Colorado QuitLine outcomes among FY2010-2011 enrollees. 2012. http://www.ucdenver.edu/academics/colleges/PublicHealth/community/CEPEG/WkProducts/Reports/Documents/71QL%20evaluation%20report,%20corrected%20final%20with%20q'aires,%202-9-12.pdf. Accessed 16 Sep 2016.

17. Stead LF, Perera R, Bullen C, et al. Nicotine replacement therapy for smoking cessation. *Cochrane Database Syst Rev.* 2012;11:CD000146.
18. Piper ME, Smith SS, Schlam TR, et al. A randomized placebo-controlled clinical trial of 5 smoking cessation pharmacotherapies. *Arch Gen Psychiatry.* 2009;66(11):1253-1262.
19. Bolt DM, Piper ME, Theobald WE, Baker TB. Why two smoking cessation agents work better than one: role of craving suppression. *J Consult Clin Psychol.* 2012;80(1):54-65.
20. Scott-Sheldon LA, Lantini R, Jennings EG, et al. Text messaging-based interventions for smoking cessation: a systematic review and meta-analysis. *JMIR mHealth and uHealth.* 2016;4(2):e49.
21. Whittaker R, McRobbie H, Bullen C, Rodgers A, Gu Y. Mobile phone-based interventions for smoking cessation. *Cochrane Database Syst Rev.* 2016;4:CD006611.
22. Cahill K, Hartmann-Boyce J, Perera R. Incentives for smoking cessation. *Cochrane Database Syst Rev.* 2015(5):CD004307.
23. Halpern SD, French B, Small DS, et al. Randomized trial of four financial-incentive programs for smoking cessation. *N Engl J Med.* 2015;372(22):2108-2117.
24. Idler EL, Benyamini Y. Self-rated health and mortality: a review of twenty-seven community studies. *J Health Soc Behav.* 1997;38(1):21-37.
25. Short ME, Goetzel RZ, Pei X, et al. How accurate are self-reports? Analysis of self-reported health care utilization and absence when compared with administrative data. *J Occup Environ Med.* 2009;51(7):786-796.
26. Tedeschi GJ, Cummins SE, Anderson CM, Anthenelli RM, Zhuang YL, Zhu SH. Smokers with self-reported mental health conditions: a case for screening in the context of tobacco cessation services. *PLoS One.* 2016;11(7):e0159127.
27. Center for Tobacco Research and Intervention. Medicaid Incentives to Prevent Chronic Disease: Wisconsin Striving to Quit – Quit Line Incentive Program Final Report. 2016. Report to the Wisconsin Department of Health Services. Report No. P-01700.
28. Sanders GD, Neumann PJ, Basu A, et al. Recommendations for conduct, methodological practices, and reporting of cost-effectiveness analyses: second Panel on Cost-Effectiveness in Health and Medicine. *JAMA.* 2016;316(10):1093-1103.
29. Feirman SP, Glasser AM, Teplitskaya L, et al. Medical costs and quality-adjusted life years associated with smoking: a systematic review. *BMC Public Health.* 2016;16:646.
30. Hayes AF. *Introduction to mediation, moderation, and conditional process analysis: A regression-based approach.* New York: The Guilford Press; 2013.
31. Rose JE, Herskovic JE, Behm FM, Westman EC. Precessation treatment with nicotine patch significantly increases abstinence rates relative to conventional treatment. *Nicotine Tob Res.* 2009;11(9):1067-1075.
32. Schuurmans MM, Diacon AH, van Biljon X, Bolliger CT. Effect of pre-treatment with nicotine patch on withdrawal symptoms and abstinence rates in smokers subsequently quitting with the nicotine patch: a randomized controlled trial. *Addiction.* 2004;99(5):634-640.
33. Fucito LM, Bars MP, Forray A, et al. Addressing the evidence for FDA nicotine replacement therapy label changes: a policy statement of the Association for the Treatment of Tobacco use and Dependence and the Society for Research on Nicotine and Tobacco. *Nicotine Tob Res.* 2014;16(7):909-914.
