# Supplementary material for: Tobacco Quitline Retreatment Interventions Among Adults With Socioeconomic Disadvantage: A Factorial Randomized Clinical Trial
Source: JAMA Netw Open. 2024 Nov 6;7(11):e2443044. doi: 10.1001/jamanetworkopen.2024.43044 (PMC11541633; doi:10.1001/jamanetworkopen.2024.43044)
Supplement: Supplement 2. — eTable 1. Participant Demographic and Tobacco Use Characteristics by Four Experimental Factors and Overall eTable 2. Intervention Delivery by Condition and by Factor Level in the 2x2x2x2 Factorial Experiment (N = 1316) eTable 3. Logistic Regression Models for 26-Week Post-Quit-Date Self-Reported 7-Day Point Prevalence Abstinence Using the Intent-to-Treat Principle With Missing Cases Coded as Smoking eTable 4. Sensitivity Analysis of Logistic Regression Models for Primary Outcome 26-Week Post-Quit-Date Biochemically Verified 7-Day Point Prevalence Abstinence Using Missing-Not-at-Random (MNAR) eTable 5. Sensitivity Analysis of Logistic Regression Models for Primary Outcome 26-Week Post-Quit-Date Biochemically Verified 7-Day Point Prevalence Abstinence Using Multiple Imputation (Missing-at-Random) eTable 6. Sensitivity Analysis of Adjusted Logistic Regression Models for Primary Outcome 26-Week Post-Quit-Date Biochemically Verified 7-Day Point Prevalence Abstinence Using the Intent-to-Treat Principle With Missing Cases Coded as Smoking eFigure 1. Biochemically Confirmed Intent-to-Treat 26-Week Post-Quit-Day Abstinence Rates by All Treatment Combinations eFigure 2. Biochemically Confirmed Intent-to-Treat 26-Week Post-Quit-Day Abstinence Rates by Counseling Calls x NRT x Financial Incentives eReferences [file jamanetwopen-e2443044-s002.pdf]

Supplementary Online Content

Kaye JT, Kirsch JA, Bolt DM, et al. Tobacco quitline retreatment interventions among adults with socioeconomic disadvantage: a factorial randomized clinical trial. *JAMA Netw Open*. 2024;7(11):e2443044. doi:10.1001/jamanetworkopen.2024.43044

eMethods 1. Sample Size, Power, and Randomization

eTable 1. Participant Demographic and Tobacco Use Characteristics by Four Experimental Factors and Overall

eTable 2. Intervention Delivery by Condition and by Factor Level in the 2x2x2x2 Factorial Experiment (N=1316)

eTable 3. Logistic Regression Models for 26-Week Post-Quit-Date Self-Reported 7-Day Point Prevalence Abstinence Using the Intent-to-Treat Principle With Missing Cases Coded as Smoking

eTable 4. Sensitivity Analysis of Logistic Regression Models for Primary Outcome 26-Week Post-Quit-Date Biochemically Verified 7-Day Point Prevalence Abstinence Using Missing-Not-at-Random (MNAR)

eTable 5. Sensitivity Analysis of Logistic Regression Models for Primary Outcome 26-Week Post-Quit-Date Biochemically Verified 7-Day Point Prevalence Abstinence Using Multiple Imputation (Missing-at-Random)

eTable 6. Sensitivity Analysis of Adjusted Logistic Regression Models for Primary Outcome 26-Week Post-Quit-Date Biochemically Verified 7-Day Point Prevalence Abstinence Using the Intent-to-Treat Principle With Missing Cases Coded as Smoking

eFigure 1. Biochemically Confirmed Intent-to-Treat 26-Week Post-Quit-Day Abstinence Rates by All Treatment Combinations

eFigure 2. Biochemically Confirmed Intent-to-Treat 26-Week Post-Quit-Day Abstinence Rates by Counseling Calls x NRT x Financial Incentives

eReferences

This supplementary material has been provided by the authors to give readers additional information about their work.

© 2024 Kaye JT et al. *JAMA Network Open.*

eMethods. Sample Size, Power, and Randomization

The study was powered to detect meaningful and feasible abstinence effect sizes for main effects and 2-way interactions. Based on our previous work in similar populations and remotely delivered treatments, we anticipated low base rates of biochemically confirmed abstinence in the low-intensity or "off" conditions; these rates were 4% and 9% in prior studies[1,2]. We estimated power for various increments in abstinence (5%, 6%, 7%, or 8%) assuming a base rate of 5%, 10%, or 15%. For these power analyses, we also assumed alpha = .05 and two-sided tests, with target power ≥ .80. Results showed that an overall sample size of 1408 (704 per group) yielded power ≥ .83 for main effects for all scenarios except one (15% base rate vs 20%; power = .695). Thus, we selected a target sample size of 704 per group we expected would be sufficiently large to detect at least small/medium-sized (7.5% of variance) effects even with measurement error.

Recruitment was slower than projected due to declining WTQL call volumes during the study period, particularly during the COVID-19 public health emergency. We closed recruitment after enrolling 1316 participants, 93.47% of the target sample of 1408 to permit completion of all follow-up activities within the funding period and before a scheduled change in WTQL programs and platforms.

Randomization occurred in blocks of 16, stratified on participant sex (male, female), racial minority status (minoritized racial group, White), and socioeconomic disadvantage criterion met for study inclusion (at the Index Quit Attempt were uninsured, Medicaid-insured, or non-Medicaid insured and no more than high school education). Race was stratified into two groups based on anticipated sample sizes of racial minority groups given that Wisconsin is less racially diverse than the US generally. There were 13 stratification categories defined by the crossing of the three stratification variables (2 x 2 x 3 = 12) and one level for participants with unknown/other gender or race. Fifteen participants were randomized based on inaccurate stratification category due to data entry error or system generated error (e.g., incorrect race/gender, insurance status used at time of enrollment rather than from Index Quit Attempt). The largest observed stratification category was Female, White, and Medicaid-insured (N=288). The initial study protocol was to stratify based on race and ethnicity. However, prior to the study launch, a separate randomization protocol was created to include the three variables implemented in the study. We determined that the number of Hispanic/Latino/a/e/x WTQL clients likely to meet recruitment prerequisites was likely to be too small to support effective stratification on this variable to randomize participants to 16 conditions, whereas the distribution based on sex would support this. We also deemed it important to balance randomization on socioeconomic disadvantage criterion that made participants eligible for study participation, as we were concerned that imbalances in insurance status, Medicaid eligibility, and education could occur without stratifying randomization to this factor.

© 2024 Kaye JT et al. *JAMA Network Open.*

eTable 1. Participant demographic and tobacco use characteristics by four experimental factors.

			Counseling		Medication		SmokefreeTXT		Financial Incentives	
		Full Sample	1 Call	4 Calls	2wk Patch	4wk Patch + Lozenge	No texts	Yes texts	No Incentive	Incentive
Variable [a]	Level	No. (%)	No. (%)	No. (%)	No. (%)	No. (%)	No. (%)	No. (%)	No. (%)	No. (%)
Gender (n=1312)	Female	760 (57.8)	383 (57.9)	378 (57.8)	376 (57.5)	385 (58.2)	376 (57.2)	385 (58.4)	380 (57.4)	381 (58.3)
	Male	552 (41.9)	278 (42.0)	275 (42.0)	278 (42.5)	275 (41.5)	280 (42.6)	273 (41.4)	281 (42.4)	272 (41.6)
Ethnicity (n=1308) [b]	Hispanic or Latino	49 (3.7)	23 (3.5)	26 (4.0)	23 (3.5)	26 (3.9)	25 (3.8)	24 (3.6)	23 (3.5)	26 (4.0)
	Not Hispanic or Latino	1259 (95.7)	637 (96.2)	622 (95.1)	626 (95.7)	633 (95.6)	629 (95.7)	630 (95.6)	633 (95.6)	626 (95.7)
Race (n=1308) [b]	Black or African American	349 (26.5)	170 (25.7)	179 (27.4)	175 (26.8)	174 (26.3)	179 (27.2)	170 (25.8)	171 (25.8)	178 (27.2)
	White or Caucasian	866 (65.8)	433 (65.4)	433 (66.2)	432 (66.1)	434 (65.6)	430 (65.4)	436 (66.2)	438 (66.2)	428 (65.4)
	Other Race	93 (7.1)	56 (8.5)	37 (5.7)	42 (6.4)	51 (7.7)	44 (6.7)	49 (7.4)	48 (7.3)	45 (6.9)
Health insurance plan type (n=1311)	Commercial	64 (4.9)	27 (4.1)	37 (5.7)	32 (4.9)	32 (4.8)	29 (4.4)	35 (5.3)	37 (5.6)	27 (4.1)
	Medicaid	756 (57.4)	381 (57.6)	375 (57.3)	379 (58.0)	377 (56.9)	378 (57.5)	378 (57.4)	381 (57.6)	375 (57.3)
	Medicare	238 (18.1)	122 (18.4)	116 (17.7)	122 (18.7)	116 (17.5)	119 (18.1)	119 (18.1)	118 (17.8)	120 (18.3)
	Uninsured	253 (19.2)	131 (19.8)	122 (18.7)	117 (17.9)	136 (20.5)	130 (19.8)	123 (18.7)	124 (18.7)	129 (19.7)
Education (n=1308)	Less than Grade 12/GED	292 (22.2)	148 (22.4)	144 (22.0)	147 (22.5)	145 (21.9)	163 (24.8)	129 (19.6)	150 (22.7)	142 (21.7)
	Grade 12 or GED	575 (43.7)	286 (43.2)	289 (44.2)	290 (44.3)	285 (43.1)	277 (42.2)	298 (45.2)	291 (44.0)	284 (43.4)
	Some College or More	441 (33.5)	226 (34.1)	215 (32.9)	213 (32.6)	228 (34.4)	214 (32.6)	227 (34.4)	217 (32.8)	224 (34.3)
Tobacco product use (n=1311)	Cigarette use only	1127 (86.0)	514 (77.6)	532 (81.3)	528 (80.7)	518 (78.2)	523 (79.6)	523 (79.4)	518 (78.2)	528 (80.7)
	Cigarette and other tobacco product use	184 (14.0)	122 (18.4)	96 (14.7)	99 (15.1)	119 (18.0)	107 (16.3)	111 (16.8)	120 (18.1)	98 (15.0)
Smokes menthol cigarettes (n=1311)	No	627 (47.6)	324 (48.9)	303 (46.3)	312 (47.7)	315 (47.6)	312 (47.5)	315 (47.8)	319 (48.2)	308 (47.1)
	Yes	684 (52.0)	336 (50.8)	348 (53.2)	342 (52.3)	342 (51.7)	342 (52.1)	342 (51.9)	340 (51.4)	344 (52.6)
Time to use tobacco after waking (n=1301)	Within 30 minutes	1118 (85.6)	507 (76.6)	505 (78.8)	482 (73.7)	540 (81.7)	514 (78.2)	508 (77.1)	515 (77.8)	507 (77.5)
	After 30 minutes	188 (14.4)	135 (20.4)	123 (18.8)	147 (22.5)	111 (16.8)	130 (19.8)	128 (19.4)	123 (18.6)	135 (20.6)
		**Mean (SD)**	**Mean (SD)**	**Mean (SD)**	**Mean (SD)**	**Mean (SD)**	**Mean (SD)**	**Mean (SD)**	**Mean (SD)**	**Mean (SD)**
Age (n=1316)		53.1 (11.9)	53.0 (11.6)	53.3 (12.3)	52.9 (11.9)	53.4 (11.9)	53.8 (11.6)	52.5 (12.2)	53.1 (11.9)	53.2 (11.9)
Motivation to quit smoking, 1-10 (n=1080) [c]		8.5 (1.7)	8.5 (1.8)	8.6 (1.7)	8.5 (1.8)	8.5 (1.7)	8.6 (1.8)	8.5 (1.7)	8.4 (1.8)	8.6 (1.7)
Cigarettes per day, 0-99 (n=1265)		18.2 (10.4)	15.3 (9.1)	14.8 (7.8)	14.9 (8.5)	15.2 (8.4)	15.1 (8.7)	15.0 (8.2)	15.1 (8.6)	15.1 (8.3)

[a] All demographic, smoking, and health characteristics were self-reported by participants at study enrollment. [b] Race and ethnicity were self-reported by clients at the time of quitline registration using the classifications in the Minimum Data Set Intake requirements specified by the North American Quitline Consortium for US quitlines. "Other Race" includes Native American/Alaska Native (14), Asian (3), Native Hawaiian/Pacific Islander (2), Arab or Arab American (2), Multi-racial (6), and self-reported "Other" response option (66). [c] Motivation to quit smoking rated from 1, not at all, to 10, extremely.

© 2024 Kaye JT et al. *JAMA Network Open.*

eTable 2. Intervention delivery by condition and by factor level in the 2x2x2x2 factorial experiment (N=1316)

Condition	*Calls Completed*			*Medication Dispensed*		*SmokefreeTXT Use* [a]		*Incentives Earned* ($)		
	Median	Mean	SD	Patch N (%)	Lozenge N (%)	Enrolled N (%)	Retained N (%)	Median	Mean	SD
**Sixteen Conditions**										
1 WTQL Call, 2 Wks Patch, No SFTXT, $0 (n=85)	1.0	1.07	0.72	70 (94.6)	0 (0)	0 (0)	0 (0)	0.00	0.00	0.00
1 WTQL Call, 2 Wks Patch, No SFTXT, $30 (n=80)	1.0	1.26	0.88	67 (94.4)	0 (0)	0 (0)	0 (0)	30.00	28.50	6.58
1 WTQL Call, 2 Wks Patch, SFTXT, $0 (n=84)	1.0	1.17	0.90	65 (94.2)	0 (0)	8 (9.5)	4 (50.0)	0.00	0.00	0.00
1 WTQL Call, 2 Wks Patch, SFTXT, $60 (n=83)	1.0	1.27	0.94	67 (90.5)	0 (0)	22 (26.5)	19 (86.4)	30.00	28.92	7.33
1 WTQL Call, 4 Wks Patch+Lozenge, No SFTXT, $0 (n=82)	1.0	1.24	0.85	69 (93.2)	67 (90.5)	0 (0)	0 (0)	0.00	0.00	0.00
1 WTQL Call, 4 Wks Patch+Lozenge, No SFTXT, $30 (n=84)	1.0	1.33	0.97	67 (89.3)	67 (89.3)	0 (0)	0 (0)	30.00	28.21	7.14
1 WTQL Call, 4 Wks Patch+Lozenge, SFTXT, $0 (n=82)	1.0	1.04	0.48	69 (93.2)	67 (90.5)	18 (21.9)	11 (61.1)	0.00	0.00	0.00
1 WTQL Call, 4 Wks Patch+Lozenge, SFTXT, $60 (n=82)	1.0	1.44	1.20	61 (84.7)	60 (83.3)	18 (21.9)	15 (83.3)	30.00	28.54	9.31
4 WTQL Calls, 2 Wks Patch, No SFTXT, $0 (n=80)	2.0	2.40	1.45	64 (94.1)	0 (0)	0 (0)	0 (0)	0.00	0.00	0.00
4 WTQL Calls, 2 Wks Patch, No SFTXT, $120 (n=81)	3.0	3.10	1.75	66 (90.4)	0 (0)	0 (0)	0 (0)	90.00	80.74	39.58
4 WTQL Calls, 2 Wks Patch, SFTXT, $0 (n=81)	2.0	2.35	1.48	62 (91.2)	0 (0)	11 (13.6)	8 (72.7)	0.00	0.00	0.00
4 WTQL Calls, 2 Wks Patch, SFTXT, $150 (n=80)	2.5	2.72	1.71	67 (94.4)	0 (0)	14 (17.5)	11 (78.6)	60.00	75.75	40.78
4 WTQL Calls, 4 Wks Patch+Lozenge, No SFTXT, $0 (n=83)	2.0	2.55	1.39	65 (85.5)	65 (8.5)	0 (0)	0 (0)	0.00	0.00	0.00
4 WTQL Calls, 4 Wks Patch+Lozenge, No SFTXT, $120 (n=82)	3.0	2.88	1.61	68 (90.7)	65 (86.7)	0 (0)	0 (0)	90	78.29	37.64
4 WTQL Calls, 4 Wks Patch+Lozenge, SFTXT, $0 (n=85)	2.0	2.65	1.62	66 (89.2)	66 (89.2)	15 (17.6)	9 (60.0)	0.00	0.00	0.00
4 WTQL Calls, 4 Wks Patch+Lozenge, SFTXT, $150 (n=82)	3.0	2.82	1.67	65 (85.5)	64 (84.2)	21 (25.6)	17 (80.9)	75.00	73.90	35.34
**Four Factors**										
1 WTQL Call (n=662) [b]	1	1.23	0.89	535 (91.8)	261 (44.8)	66 (9.9)	49 (74.2)	0.00	14.18	15.26
4 WTQL Calls (n=654)	3	2.68	1.60	523 (90.0)	260 (44.8)	61 (9.3)	45 (73.8)	0.00	38.35	47.10
2 Wks Patch (n=654) [c]	1	1.91	1.49	528 (93.0)	0 (0)	55 (8.4)	42 (76.4)	0.00	26.42	37.73
4 Wks Patch+Lozenge (n=662)	1	2.00	1.48	530 (88.9)	521 (87.4)	72 (10.9)	52 (72.2)	0.00	25.97	36.17
No SFTXT (n=657) [d]	1	1.97	1.47	536 (91.5)	264 (45.1)	0 (0.0)	0 (0.0)	0.00	26.80	37.82
SFTXT (n=659)	1	1.93	1.49	578 (90.3)	257 (44.5)	127 (19.3)	94 (74.0)	0.00	25.58	30.06
$0 (n=662) [e]	1	1.81	1.36	530 (91.9)	265 (45.9)	52 (7.9)	32 (61.5)	0.00	0.00	0.00
$30-$150 (n=654)	1	2.10	1.59	528 (89.9)	256 (43.6)	75 (11.5)	62 (82.7)	30.00	52.70	36.72

[a] SmokefreeTXT retention was defined as not texting "STOP" to stop receiving text messages prior to 6 weeks after their target quit date.
[b] Four-proactive calls significantly increased the number of counseling calls completed versus the 1-call condition, b = 1.5, 95% CI 1.3 to 1.6, $t(1314) = 20.44$, $p < .001$.
[c] Provision of 4 weeks of combination NRT resulted in higher rates of nicotine lozenge distribution, $\chi^2(1) = 898.8$, $p < .001$, but lower rates of nicotine patch distribution, $\chi^2(1) = 5.7$, $p = .017$, versus 2 weeks of nicotine patch provision.

© 2024 Kaye JT et al. *JAMA Network Open.*

[d] The SmokefreeTXT factor increased the percentage of participants who enrolled in the SmokefreeTXT program, $\chi^2(1) = 137.9$, $p < .001$. Data provided by SmokefreeTXT from participants who enrolled with study shortcode.
[e] Participants earned significantly more financial incentives in the incentive condition versus the no-incentive condition, $b = 52.71$, 95% CI 49.91 to 55.51, $t(1314) = 36.9$, $p < .001$.

© 2024 Kaye JT et al. *JAMA Network Open.*

eTable 3. Logistic regression models for 26-week post-quit-day self-reported 7-day point prevalence abstinence using the intent-to-treat principle with missing cases coded as smoking.

**Main Effects**	**B**	**SE**	**Wald**	**P**	**OR (95% CI)**
Counseling	.070	0.063	1.227	.27	1.07 [0.95 - 1.21]
NRT	.065	0.063	1.079	.30	1.07 [0.94 - 1.21]
SmokefreeTXT	-.058	0.063	0.855	.36	0.94 [0.83 - 1.07]
Incentive	-.056	0.063	0.796	.37	0.95 [0.84 - 1.07]
**2-way Interactions**					
Counseling x NRT	.061	0.063	0.932	.33	1.06 [0.94 - 1.20]
Counseling x SmokefreeTXT	.021	0.063	0.117	.73	1.02 [0.90 - 1.16]
Counseling x Incentive	.101	0.063	2.562	.11	1.11 [0.98 - 1.25]
NRT x SmokefreeTXT	-.082	0.063	1.710	.19	0.92 [0.81 - 1.04]
NRT x Incentive	-.083	0.063	1.743	.19	0.92 [0.81 - 1.04]
SmokefreeTXT x Incentive	.014	0.063	0.052	.82	1.01 [0.90 - 1.15]
**3-way Interaction**					
Counseling x NRT x SmokefreeTXT	.017	0.063	0.072	.79	1.02 [0.90 - 1.15]
**Counseling x NRT x Incentive***	**.125**	**0.063**	**3.949**	**.047**	**1.13 [1.00 - 1.28]**
**Counseling x SmokefreeTXT x Incentive***	**-.210**	0.063	**11.110**	**<.001**	**0.81 [0.72 - .92]**
NRT x SmokefreeTXT x Incentive	-.011	0.063	0.029	.87	0.99 [0.88 - 1.12]
**4-way Interaction**					
Counseling x NRT x SmokefreeTXT x Incentive	.063	0.063	1.009	.32	1.07 [0.94 - 1.21]

Note: Intent-to-treat analyses were used including all participants in the analysis under the assumption that missing data reflect smoking (coded as 0 = smoking, 1 = abstinent). Effects coding were used for the main effects and all higher-order interactions (levels coded as -1 for control and +1 for active enhancement). Model covariates include 12 binary coded variables representing the observed stratification categories (the most frequent category = Female, White, and Medicaid insured was used as the reference); the model was not adjusted for any other covariates.
* $p < .05$

© 2024 Kaye JT et al. *JAMA Network Open.*

eTable 4. Sensitivity analysis of logistic regression models for primary outcome 26-week post-quit-day biochemically verified 7-day point prevalence abstinence using multiple imputation missing-not-at-random (MNAR).

**Main Effects**	**B**	**SE**	**P**	**OR (95% CI)**
Counseling	.064	0.093	.49	1.07 (0.89 - 1.28)
NRT	.115	0.087	.19	1.12 (.095 - 1.33)
SmokefreeTXT	-.106	0.091	.24	0.90 (0.75 - 1.11)
Incentive	-.097	0.093	.30	0.91 (0.76 - 1.09)
**2-way Interactions**				
Counseling x NRT	.056	0.094	.55	1.06 (0.88 - 1.27)
Counseling x SmokefreeTXT	.012	0.088	.90	1.01 (0.85 - 1.20)
Counseling x Incentive	.070	0.092	.45	1.07 (0.90 - 1.29)
NRT x SmokefreeTXT	-.120	0.093	.20	0.89 (0.74 - 1.07)
NRT x Incentive	.023	0.086	.79	1.02 (0.86 - 1.21)
SmokefreeTXT x Incentive	-.036	0.092	.70	0.97 (0.81 - 1.16)
**3-way Interaction**				
Counseling x NRT x SmokefreeTXT	.022	0.089	.80	1.02 (0.86 - 1.22)
**Counseling x NRT x Incentive ***	**.238**	**0.091**	**.009**	**1.27 (1.06 - 1.52)**
Counseling x SmokefreeTXT x Incentive	-.118	0.095	.22	0.89 (0.74 - 1.07)
NRT x SmokefreeTXT x Incentive	.089	0.091	.33	1.09 (0.91 - 1.31)
**4-way Interaction**				
**Counseling x NRT x SmokefreeTXT x Incentive ***	**.183**	**0.091**	**.044**	**1.20 (1.01 - 1.43)**

Note: Multiple imputation analyses were used combined with missing-not-at-random (MNAR) assumption that missing data decreases the log-odds of abstinence by 1.0[3]. Effects coding were used for the main effects and all higher-order interactions (levels coded as -1 and +1). Model covariates include 12 binary coded variables representing the observed stratification categories (the most frequent category = Female, White, and Medicaid insured was used as the reference); the model was not adjusted for any other covariates. Models using multiple imputation do not yield a Wald value.
* $p < .05$

© 2024 Kaye JT et al. *JAMA Network Open.*

eTable 5. Sensitivity analysis of logistic regression models for primary outcome 26-week post-quit-day biochemically verified 7-day point prevalence abstinence using multiple imputation (missing-at-random).

**Main Effects**	**B**	**SE**	**P**	**OR (95% CI)**
Counseling	.021	0.086	.81	1.02 (0.86 - 1.21)
NRT	.084	0.089	.35	1.09 (0.91 - 1.30)
SmokefreeTXT	-.087	0.094	.36	0.92 (0.76 - 1.11)
Incentive	-.114	0.099	.26	0.89 (0.73 - 1.09)
**2-way Interactions**				
Counseling x NRT	.030	0.087	.73	1.03 (0.87 - 1.22)
Counseling x SmokefreeTXT	.013	0.086	.88	1.01 (0.86 - 1.20)
Counseling x Incentive	.108	0.086	.21	1.11 (0.94 - 1.32)
NRT x SmokefreeTXT	-.100	0.086	.25	0.91 (0.76 - 1.07)
NRT x Incentive	-.013	0.080	.85	0.99 (0.84 - 1.15)
SmokefreeTXT x Incentive	-.037	0.083	.65	0.96 (0.82 - 1.13)
**3-way Interaction**				
Counseling x NRT x SmokefreeTXT	.004	0.094	.96	1.00 (0.83 - 1.21)
**Counseling x NRT x Incentive ***	**.221**	**0.102**	**.035**	**1.25 (1.02 - 1.53)**
Counseling x SmokefreeTXT x Incentive	-.125	0.092	.18	0.88 (0.74 - 1.06)
NRT x SmokefreeTXT x Incentive	.107	0.091	.24	1.11 (0.93 - 1.33)
**4-way Interaction**				
Counseling x NRT x SmokefreeTXT x Incentive	.141	0.092	.13	1.15 (0.96 - 1.38)

Note: Multiple imputation analyses were used under the assumption of Missing-At-Random. Effects coding were used for the main effects and all higher-order interactions (levels coded as -1 and +1). Model covariates include 12 binary coded variables representing the observed stratification categories (the most frequent category = Female, White, and Medicaid insured was used as the reference); the model was not adjusted for any other covariates. Models using multiple imputation do not yield a Wald value.
* $p < .05$

© 2024 Kaye JT et al. *JAMA Network Open.*

eTable 6. Sensitivity analysis of adjusted logistic regression models for primary outcome 26-week post-quit-day biochemically verified 7-day point prevalence abstinence using the intent-to-treat principle with missing cases coded as smoking.

Main Effects	B	SE	Wald	P	OR (95% CI)
Counseling	.035	0.090	0.151	0.70	1.04 (0.87 - 1.24)
NRT	.122	0.090	1.829	0.18	1.13 (0.95 - 1.35)
SmokefreeTXT	-.119	0.091	1.738	0.19	0.89 (0.73 - 1.06)
Incentive	-.079	0.090	0.757	0.38	0.92 (0.77 - 1.10)
**2-way Interactions**					
Counseling x NRT	.060	0.090	0.446	0.50	1.06 (0.89 - 1.27)
Counseling x SmokefreeTXT	.040	0.090	0.198	0.66	1.04 (0.87 - 1.24)
Counseling x Incentive	.029	0.090	0.102	0.75	1.03 (0.86 - 1.23)
NRT x SmokefreeTXT	-.106	0.090	1.366	0.24	0.90 (0.75 - 1.07)
NRT x Incentive	.043	0.090	0.222	0.64	1.04 (0.87 - 1.25)
SmokefreeTXT x Incentive	-.048	0.090	0.287	0.59	0.95 (0.80 - 1.14)
**3-way Interaction**					
Counseling x NRT x SmokefreeTXT	.017	0.090	0.036	0.85	1.02 (0.85 - 1.22)
**Counseling x NRT x Incentive ***	**.227**	**0.090**	**6.311**	**0.012**	**1.26 (1.05 - 1.50)**
Counseling x SmokefreeTXT x Incentive	-.139	0.090	2.374	0.12	0.87 (0.73 - 1.04)
NRT x SmokefreeTXT x Incentive	.077	0.090	0.728	0.39	1.08 (0.91 - 1.29)
**4-way Interaction**					
**Counseling x NRT x SmokefreeTXT x Incentive ***	**.191**	**0.090**	**4.448**	**0.035**	**1.21 (1.01 - 1.45)**

Note: Intent-to-treat analyses were used including all participants in the analysis under the assumption that missing data reflect smoking (coded as 0 = smoking, 1 = abstinent). Effects coding were used for the main effects and all higher-order interactions (levels coded as -1 for control and +1 for active enhancement). Model covariates include 12 binary coded variables representing the observed stratification categories (the most frequent category = Female, White, and Medicaid insured was used as the reference). Additional model covariates include 3 *a priori* selected adjustment variables: age (years), social deprivation index (SDI) score, and longest past quit attempt. Sex and race were not included as separate model covariates as they were indexed using the stratification variables.

* $p < .05$

© 2024 Kaye JT et al. *JAMA Network Open.*

eFigure 1. Biochemically confirmed intent-to-treat abstinence rates 26-weeks post-target-quit-day by all treatment combinations.

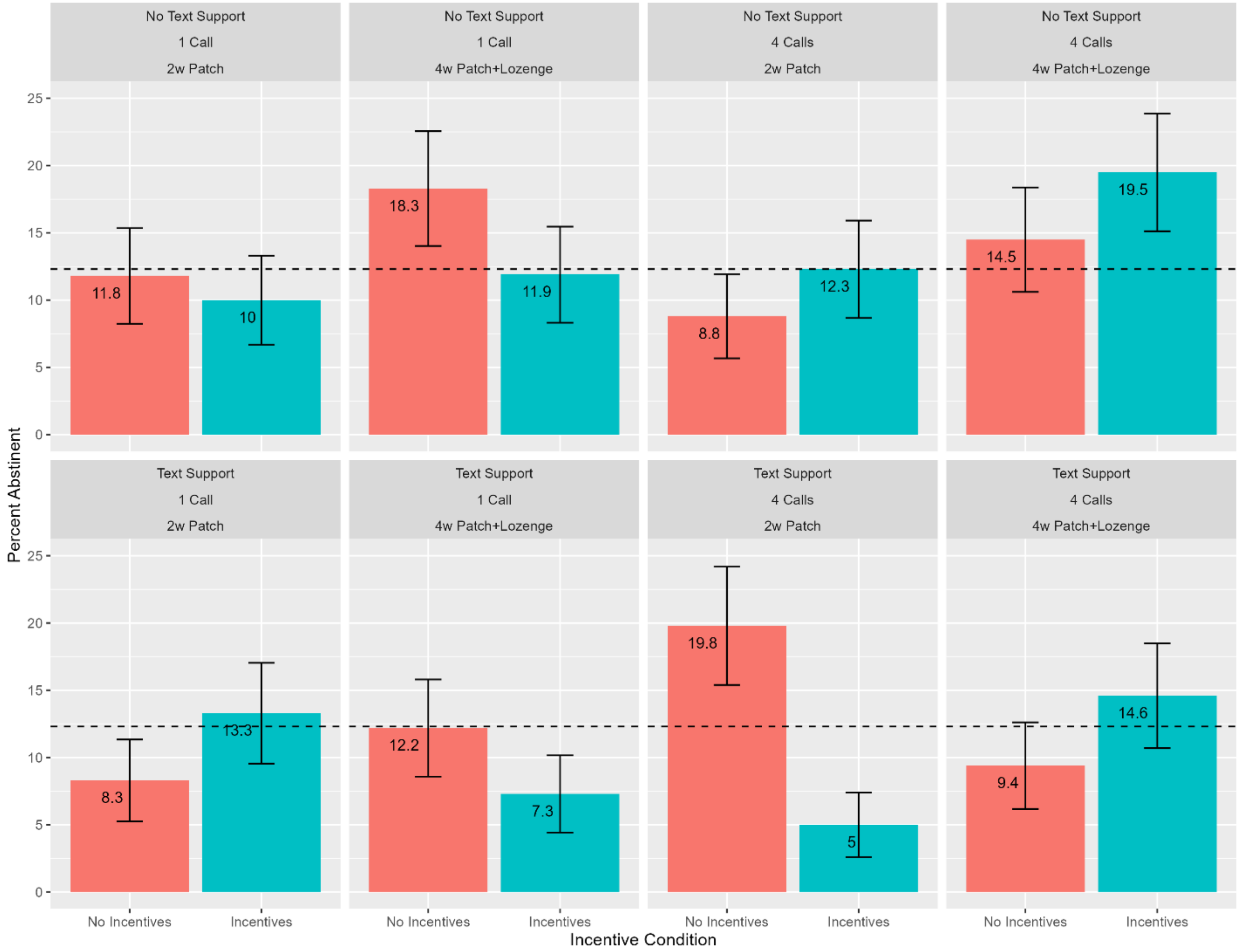

Note: Primary outcome of 26 week biochemically verified abstinence representing the full crossing of all four factors resulting in 16 conditions. Error bars represent approximate standard errors defined as $\sqrt{[p * (1-p) / n)]}$, where p is the percent abstinent and n is the number of participants that contributed to the bar. Error bars are approximate given the assumption of independent observations. Factorial data were modeled with higher order interactions so all lower-order effects are dependent on the level of the other factors. Dashed line represents the mean biochemically confirmed intent-to-treat abstinence rate (12.3%) across all conditions.

© 2024 Kaye JT et al. *JAMA Network Open.*

eFigure 2. Biochemically confirmed intent-to-treat abstinence rates 26-weeks post-target-quit-day by Counseling Calls x NRT x Financial Incentives.

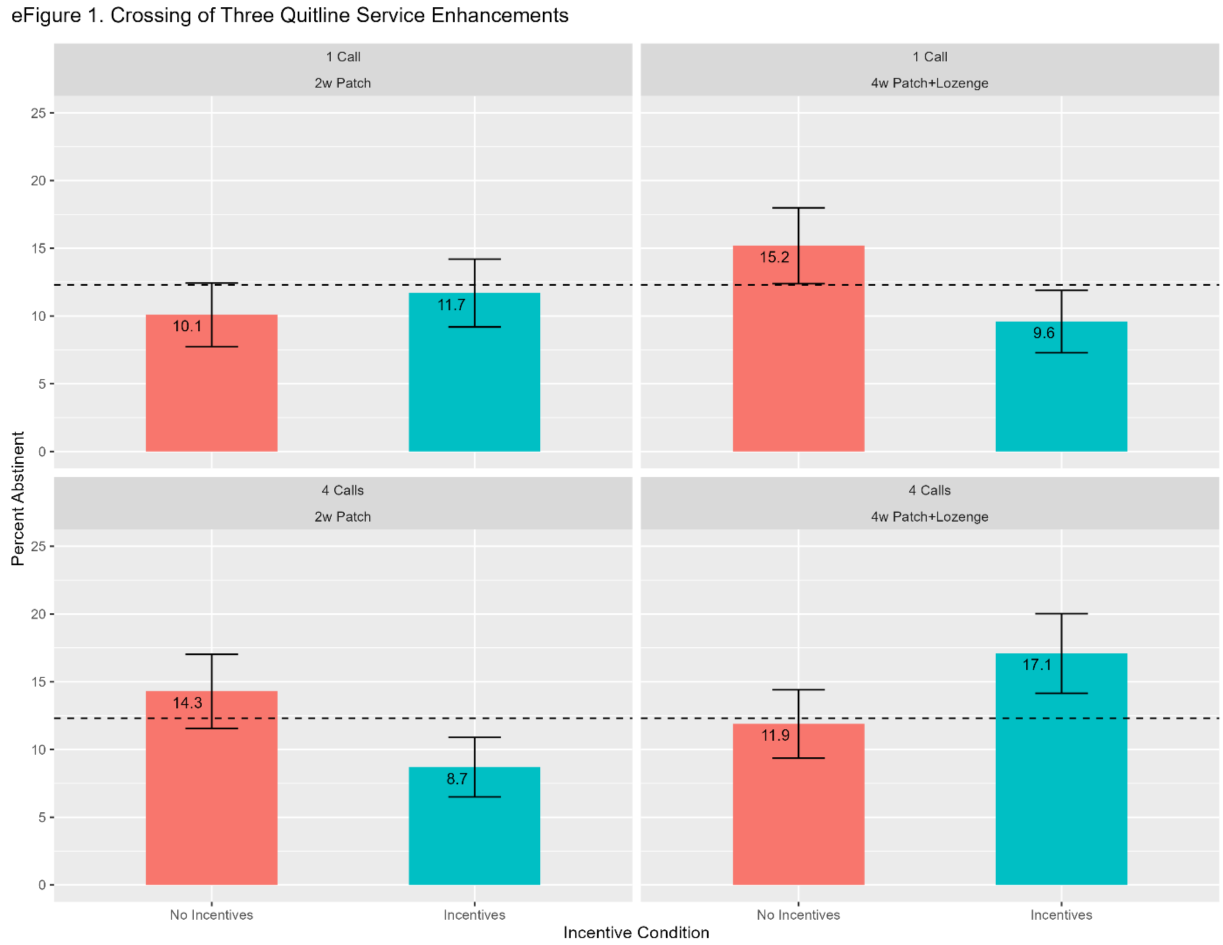

Note: Primary outcome of 26 week biochemically verified abstinence representing the effects of each of the three factors, Counseling Calls x NRT x Financial Incentives, collapsed across SmokefreeTXT factor. No clear pattern emerged as definitively driving this interaction or identifying the ideal combination of interventions. Visual inspection of the group means suggests that the combination of increased counseling calls, extended duration combination-NRT, and incentives led to relatively higher abstinence rates (17.1%). However, relatively small samples sizes limit confidence in any simple effects comparisons and would warrant replication in an independent sample. There was a significant 3-way interaction in the main analysis between Counseling Calls x NRT x Financial Incentives (Table 2). Error bars represent approximate standard errors defined as $\sqrt{[p * (1-p) / n)]}$, where p is the percent abstinent and n is the number of participants that contributed to the bar. Error bars are approximate given the assumption of independent observations. Dashed line represents the mean biochemically confirmed intent-to-treat abstinence rate (12.3%) across all conditions.

© 2024 Kaye JT et al. *JAMA Network Open.*

eReferences

1. Fraser DL, Fiore MC, Kobinsky K, et al. A randomized trial of incentives for smoking treatment in Medicaid members. *Am J Prev Med*. Dec 2017;53(6):754-763. doi:10.1016/j.amepre.2017.08.027
2. Smith SS, Keller PA, Kobinsky KH, et al. Enhancing tobacco quitline effectiveness: identifying a superior pharmacotherapy adjuvant. *Nicotine Tob Res*. Mar 2013;15(3):718-28. doi:10.1093/ntr/nts186
3. Hedeker D, Mermelstein RJ, Demirtas H. Analysis of binary outcomes with missing data: missing = smoking, last observation carried forward, and a little multiple imputation. *Addiction*. Oct 2007;102(10):1564-73.

© 2024 Kaye JT et al. *JAMA Network Open.*
