# Supplementary material for: Tobacco Quitline Retreatment Interventions Among Adults With Socioeconomic Disadvantage: A Factorial Randomized Clinical Trial
Source: JAMA Netw Open. 2024 Nov 6;7(11):e2443044. doi: 10.1001/jamanetworkopen.2024.43044 (PMC11541633; doi:10.1001/jamanetworkopen.2024.43044)
Supplement: Supplement 3. — Data Sharing Statement [file jamanetwopen-e2443044-s003.pdf]

Data Sharing Statement

Kaye. Tobacco Quitline Retreatment Interventions Among Adults With Socioeconomic Disadvantage. *JAMA Netw Open*. Published November 06, 2024.
doi:10.1001/jamanetworkopen.2024.43044

Data
Additional Information: ClinicalTrials.gov Identifier NCT03538938
Data available: No

Additional Information
Explanation for why data not available: Participants did not provide informed consent for public sharing of their data. Given that the study population was recruited specifically based on characteristics that confer socioeconomic disadvantage and the potentially stigmatizing nature of the information collected, data from this study will not be shared in an open-access repository. Qualified researchers or other individuals may send data access requests to the corresponding author (Danielle McCarthy), which may be approved under a Data Use Agreement with the University of Wisconsin.
